# Unraveling the parahormetic mechanism underlying the health-protecting effects of grapeseed procyanidins

**DOI:** 10.1016/j.redox.2023.102981

**Published:** 2023-12-07

**Authors:** G. Baron, A. Altomare, L. Della Vedova, F. Gado, O. Quagliano, S. Casati, N. Tosi, L. Bresciani, D. Del Rio, G. Roda, A. D'Amato, C. Lammi, A. Macorano, S. Vittorio, G. Vistoli, L. Fumagalli, M. Carini, A. Leone, M. Marino, C. Del Bo’, G. Miotto, F. Ursini, P. Morazzoni, G. Aldini

**Affiliations:** a Department of Pharmaceutical Sciences (DISFARM), Università degli Studi di Milano, Via Mangiagalli 25, 20133, Milan, Italy; b Department of Biomedical, Surgical and Dental Sciences, Università degli Studi di Milano, Via Luigi Mangiagalli 37, 20133, Milan, Italy; c Human Nutrition Unit, Department of Food & Drug, University of Parma, Via Volturno 39, 43125, Parma, Italy; d International Center for the Assessment of Nutritional Status and the Development of Dietary Intervention Strategies (ICANS-DIS), Via Sandro Botticelli 21, 20133, Milan, Italy; e Department of Food, Environmental and Nutritional Sciences (DeFENS), Division of Human Nutrition, Università degli Studi di Milano, Via Luigi Mangiagalli 25, 20133, Milan, Italy; f Department of Molecular Medicine, Viale G. Colombo, 3, University of Padova, 35121, Padova, Italy; g Divisione Nutraceutica, Distillerie Umberto Bonollo S.p.A, 35035, Mestrino, Italy

**Keywords:** (Poly)Phenols, Proanthocyanidins, 5-(3′,4′-dihydroxyphenyl)-γ-valerolactone, Anti-inflammatory and antioxidant activities, Metabolism, Microbiota, Nrf2, NFκB, Keap1

## Abstract

Proanthocyanidins (PACs), the predominant constituents within Grape Seed Extract (GSE), are intricate compounds composed of interconnected flavan-3-ol units. Renowned for their health-affirming properties, PACs offer a shield against a spectrum of inflammation associated diseases, such as diabetes, obesity, degenerations and possibly cancer. While monomeric and dimeric PACs undergo some absorption within the gastrointestinal tract, their larger oligomeric and polymeric counterparts are not bioavailable. However, higher molecular weight PACs engage with the colonic microbiota, fostering the production of bioavailable metabolites that undergo metabolic processes, culminating in the emergence of bioactive agents capable of modulating physiological processes.

Within this investigation, a GSE enriched with polymeric PACs was employed to explore in detail their impact. Through comprehensive analysis, the present study unequivocally verified the gastrointestinal-mediated transformation of medium to high molecular weight polymeric PACs, thereby establishing the bioaccessibility of a principal catabolite termed 5-(3′,4′-dihydroxyphenyl)-γ-valerolactone (VL). Notably, our findings, encompassing cell biology, chemistry and proteomics, converge to the proposal of the notion of the capacity of VL to activate, upon oxidation to the corresponding quinone, the nuclear factor E2-related factor 2 (Nrf2) pathway—an intricate process that incites cellular defenses and mitigates stress-induced responses, such as a challenge brought by TNFα. This mechanistic paradigm seamlessly aligns with the concept of para-hormesis, ultimately orchestrating the resilience to stress and the preservation of cellular redox equilibrium and homeostasis as benchmarks of health.

## 1 Introduction

Proanthocyanidins (PACs), a class of secondary metabolites widely distributed among plants, are oligomers (polymerization degree, n = 2–4) and polymers (n ≥ 5) of flavan-3-ols characterized by two aromatic rings linked through a three-carbon chain. The building blocks include catechin/epicatechin and gallocatechin/epigallocatechin [1,2].

PACs have shown different health-protecting effects. In the gastro-intestinal (GI) tract, PACs have the potential to contribute to the host's defense against pathogens, decrease the inflammatory and pro-oxidant processes occurring in the gastric and colonic mucosa, favoring ulcer healing and contributing to the mucosa integrity and reducing the risk of colorectal cancer [3]. In the small intestine, they modulate the secretion of GI hormones, the epithelial transport of water and electrolytes, and the GI transit [4,5]. Besides acting locally at the GI tract, the biological effects of PACs extend also to the systemic level, where an activity as neuroprotectants and anti-obesity, anti-carcinogenesis, anti-diabetes, and anti-inflammatory has been reported [[6], [7], [8]].

Monomeric flavan-3-ols and dimeric PACs can be partially absorbed in the GI tract, with the bioavailability of dimeric PACs being 100 times lower than that of monomeric forms [9,10]. Orally ingested oligomeric and polymeric PACs, instead, are not bioavailable [11] but can interact with the microbiota in the colon. This results in the production of catabolites through fission of the 5 C-ring, *e.g*. into phenyl-γ-valerolactones and hydroxyphenylvaleric acids, which can be subsequently degraded into low molecular weight phenolic acids and aromatic compounds [[12], [13], [14]]. There is some evidence that these phenolic catabolites are bioavailable and in turn subjected to phase II metabolism at hepatocyte level, to produce conjugated derivatives that can be detected in urine [15]. These compounds are therefore potentially available to target tissues and organs influencing (patho)physiological scenarios.

Based on the above-mentioned premises, the aim of the present work was to confirm, validate and expand the comprehensive pathway underlying the health-protecting effects of PACs. An innovative standardized grape seed extract (GSE) featuring low monomeric catechin and epicatechin content and a high concentration of oligo-polymeric procyanidins was used [16]. We first confirmed the GI and systemic metabolic conversion of PACs by using an integrated *in vitro* and *in vivo* (placebo-controlled intervention study) approach. 5-(3′,4′-dihydroxyphenyl)-γ-valerolactone (VL) was recognized as the main PACs catabolite produced by colonic microbiota. We further validated the notion that VL is bioavailable as evidenced by identifying the corresponding phase II metabolites (sulfate and glucuronide) in the urine of volunteers treated with GSE. Finally, cell biology evidence integrated by proteomic analyses brought further support to the leading hypothesis that VL produced from PACs operates, following a redox transition to quinone, as an electrophilic activator of the nuclear factor E2-related factor 2 (Nrf2). Therefore, PACs emerge as a xenobiotic phytochemical that bestow a health-protective effect achieved through the contribution to the activation of cell-protective, anti-stress mechanisms, primarily involving the Nrf2 pathway [17]. This mechanism fits into the broader framework of para-hormetic effects [18], collectively aiding the preservation of redox steady-state impacting on cellular homeostasis [19].

## 2 Materials and methods

### 2.1 Chemicals

The grape seed extract used in this study (named with the acronymous GSE within the text), was prepared by Distillerie Bonollo Umberto S.p.A (Mestrino, PD, Italy; commercial name of the extract, Ecovitis®) using selected seeds obtained from Northeast Italian wineries and an extractive food-related procedure based on the sequential combination of an aqueous-infusion and tangential-flow filtration with membranes with varying degrees of selective porosity. The extract was characterized by low monomeric catechin and epicatechin content and a high concentration of oligo-polymeric procyanidins as already reported [16].

All the other chemicals are listed in Supplementary Information (S.0).

### 2.2 *In vitro* fermentation of grape byproduct fractions and UHPLC-MS/MS analysis of fecal catabolites

The analysis of PACs contained in grape seed extract was performed in fraction at different degrees of polymerization isolated by high-performance gel permeation chromatography [20] and detailed in the SI (S.1.1, S.1.2, and S.1.3). The *in vitro* fecal fermentation of grape seed extract fractions was performed following previously published data [[21], [22], [23], [24], [25]]. Details about chemicals, reagents and procedure are given in SI (S.2.1). Native flavan-3-ols and phenolic catabolites generated during the *in vitro* fermentation were extracted adopting the method previously reported by Di Pede et al. [23], with slight modifications (S.2.2). Then, the fecal extracts were analyzed through high-performance liquid chromatography coupled with tandem mass spectrometry (UHPLC-ESI-MS/MS) [23] (S.2.3). Chromatographic and ionization parameters were based on the method of Brindani et al. [26]. A total of 76 compounds (14 parent compounds and 62 gut microbiota catabolites) were monitored through selective reaction monitoring (SRM) mode. Quantification was performed with calibration curves of standards, when available [26,27], or using the most structurally similar compound (S.2.-Table 1). Data processing was performed using Xcalibur software (Thermo Scientific Inc., Waltham, MA, USA).

### 2.3 Intervention study

#### 2.3.1 Subject selection

Twelve healthy volunteers (both sexes, age 25–35 years old, BMI 18–25 kg/m^2^) were recruited from the population of the University of Milan. Subjects were selected based on their dietary habits evaluated through a food diary and an interview in order to ensure a homogeneous population, in particular for fruit and vegetable consumption. In addition, their eligibility was assessed by a physician through a routine medical examination. Subjects were included whether healthy, non-smokers, no history of diseases (e.g., renal insufficiency, chronic constipation, diarrhea, or any other gastrointestinal disorder) and no allergy to grape or grape-derived products. Subjects were excluded if taking drugs, medications, or supplements for at least one month before the beginning of the study. Subjects were also excluded whether they followed a specific diet (e.g., vegetarian, vegan, or macrobiotic). All participants signed the informed consent. The study was approved by the Ethics Committee of the University of Milan (September 15, 2021, Annex7, document number: 94/21), and the protocol registered at ISRCTN registry: ISRCTN39867491 (https://doi.org/10.1186/ISRCTN39867491).

#### 2.3.2 Experimental design

The study was a randomized, crossover, double-blind, placebo-controlled trial. A researcher not involved in the trial was appointed to allocate subjects (block randomization) to the different treatments. Subjects were instructed to maintain their dietary and lifestyle habits as declared before enrolment, apart from the consumption of (poly)phenol-rich foods. In particular, subjects were asked to follow a low (poly)phenol diet (e.g., no apple, berries, chocolate, coffee, tea, wine, and fruit juice) starting from 3 days before the intervention, until the end of the experiment. A list of foods allowed and not permitted was also provided to the subjects and the compliance was checked also through a 3-day food diary.

**Fig. 1:**
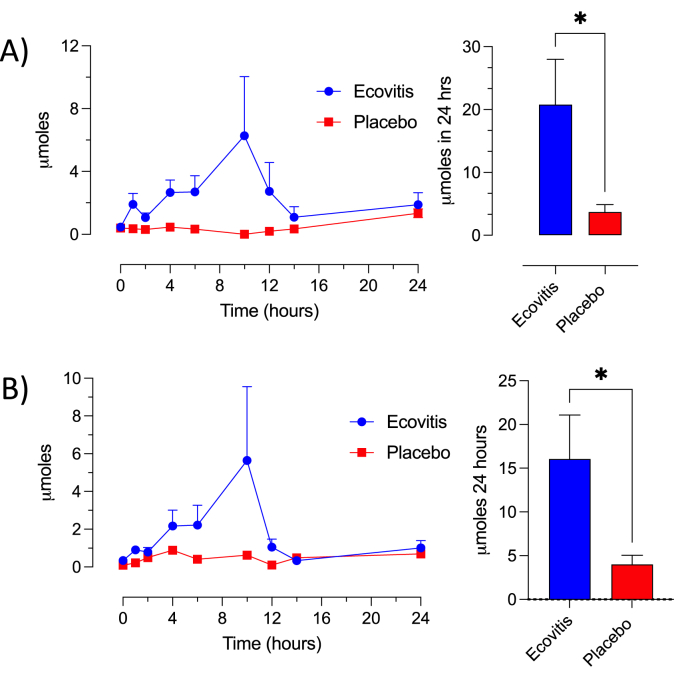
Urine excretion of 5-(hydroxyphenyl)-γ-valerolactone-sulfate (co-elution of 3′- and 4′-sulfate) (panel A) and of 5-(hydroxyphenyl)-γ-valerolactone-glucuronide (panel B). Error bars indicate standard error of mean (s.e.m.). *p < 0,05.

For the experiments, volunteers received, in a random sequence, the treatment with GSE (2 capsules/day, 150mg/capsule) or placebo (2 capsules/day, dibasic calcium phosphate 250 mg/capsule) for 7 days. Capsules were taken one in the morning and one in the evening with a glass of water. The compliance was checked by an interview. Each treatment was separated by one week of wash-out period. At the beginning and at the end of each experimental period (before and after placebo and/or GSE supplement), subjects came to the laboratories of the ICANS-DIS (University of Milan) for the analyses. Urine was collected early in the morning after an overnight fast and a standardize dinner. Successively, subjects were instructed to take another capsule (placebo and/or GSE) and collect urine. Urine collection was scheduled before the beginning of the treatments and 1, 2, 4, 6, 10, 12, 14, 24 and 48 h after the last capsule intake. The schematic representation of the pilot study is reported in S.3.-Fig. 1.

#### 2.3.3 Urinary and plasma metabolites identification by LC-MS

##### 2.3.3.1 Urine samples preparation

Urine samples were spiked with the internal standard (IS) [5-(3′,4′-dihydroxyphenyl)-γ-valerolactone D_4_] and stored at −80 °C until analysis.

An aliquot (1 mL) of urine at each time point was extracted on Thermo Scientific™ HyperSep™ C18 cartridges, dried under vacuum and diluted with 200 μL of 95 % mobile phase A (H_2_O-0.2 % HCOOH, %v/v) and 5 % mobile phase B (CH_3_CN-0.2 % HCOOH, %v/v). Each sample was analyzed in triplicate. A mixture of the three valerolactone standards (200 μM) including 5-(3′,4′-dihydroxyphenyl)-γ-valerolactone, 5-(3′-hydroxyphenyl)-γ-valerolactone-4′-sulfate and 5-(5′-hydroxyphenyl)-γ-valerolactone-3′-glucuronide was used to build the calibration curve samples. Six concentrations of the mixture in the range 0.1–20 μM were prepared in blank urine and samples were processed as previously described. Each calibration curve was obtained by plotting the peak area ratio of metabolite/IS versus the concentration of the metabolite by weighted (1/x^2^) least-squares linear regression.

##### 2.3.3.2 LC-MS/MS conditions

Each sample (5 μL) was injected into a reversed-phase Agilent Zorbax SB-C18 (150 × 2.1 mm, i.d. 3.5 μm, CPS analitica, Milan, Italy) protected by an Agilent Zorbax guard column, kept at 40 °C. The chromatographic separation was performed by a Exion LC 100 system (AB Sciex, Milan, Italy) connected to an API 4000 triple quadrupole mass spectrometer (AB Sciex, Milan, Italy), equipped with a TurboV electrospray interface (AB Sciex, Milan, Italy), operating in negative ion mode. Chromatographic and MS conditions are detailed in S.4-Table 1.

### 2.4 Cellular studies

#### 2.4.1 Anti-inflammatory and antioxidant activity in gene-reporter cells

Anti-inflammatory and antioxidant assays were performed by using two different *in vitro* cellular models [28,29]: HEK293 with the cell reporter for Nrf2 activation and R3/1 with cell reporter for NF-κB. Experimental conditions and analytical procedure are reported in S.5.1.

#### 2.4.2 Anti-inflammatory activity in human intestinal Caco-2 cells

Human Caco-2 cells were obtained from INSERM (Paris, France) and routinely sub-cultured at 50 % density and kept at 37 °C in a 5 % CO_2_ atmosphere in DMEM containing 25 mM of glucose, 3.7 g/L of NaHCO_3_, 4 mM of stable l-glutamine, 1 % nonessential amino acids, 100 U/L of penicillin, and 100 μg/L of streptomycin (complete medium), supplemented with 10 % heat-inactivated FBS. The anti-inflammatory effects of VL on the NFκB and phosphorylated (p)(ser276)-NFκB protein levels were assessed by Western blot. MTT assay was carried out following the method of Lammi C. et al. [30] and detailed in S.5.2.

### 2.5 Statistical analysis

For *in vitro* fermentation studies, a one-way ANOVA with Tukey's post-hoc test was applied to detect differences in concentration of the parent compounds and catabolites among different incubation times (T0, T5, T24) for each fraction. Differences were considered significant at p-value <0.05. ANOVA was performed using IBM SPSS Statistics version 26 (IBM, Chicago, IL, USA).

For cell studies a one-way ANOVA followed by Tukey's post-hoc analysis (GraphPad Software 9, San Diego, CA, USA) was performed. Values were expressed as means ± standard deviation (S.D.); p-values ≤0.05 were considered to be significant.

A *t*-test analysis was used to compare the amounts of metabolites excreted within 24 h in the urine of people receiving GSE with respect to placebo. Differences were considered significant for p ≤ 0.05. Data relative to urine excretion are reported as mean ± S.E.M.

### 2.6 Proteomic studies

#### 2.6.1 LFQ analysis - experimental design

Each condition tested was cultivated in biological triplicate. In detail, at the beginning of the experiment (D0), 8x10^4^ cells/well were seeded in 6-well plates and incubated at 37 °C under 5 % CO_2_ atmosphere. After 24 h (D1), once 50 % confluence was reached, the cells were treated according to the planned experimental design as shown in SI (S.6-Fig. 1). Overall, the conditions chosen were: **(i)** Control (CTR), *i.e*. untreated cells; **(ii)** VL treatment, referred to cells underwent a 48 h treatment with 75 μM VL; **(iii)** Inflammation condition achieved treating cells for 24 h with 15 ng/mL TNFα; **(iv)** VL pre-treatment (TNFα-VL) of inflamed cells consisting in the 24 h pre-treatment with 75 μM VL and then for further 24 h with 15 ng/mL TNFα. Proteolytic digestion was carried out according to the optimized procedure as reported by Ferrario et al. [28] with minor modifications as reported in S.6.2 and peptides sequencing by nano-liquid chromatography (LC) in-line tandem mass spectrometry (MS/MS) using the following analytical platform: Dionex Ultimate 3000 nano-LC system (Sunnyvale, CA, USA) coupled to the Orbitrap Fusion Tribrid mass spectrometer (Thermo Fisher Scientific, Bremen, Germany), equipped with a nano-electrospray ion source, overall set as reported by Aiello et al. [31].

#### 2.6.2 Data analysis

In label-free quantitative analysis, a key step is to analyze experimental raw LC-MS data files, and to determine the relative quantities in different samples of the observed ion species (log2 Fold-Ratio); raw files (3 biological x 3 technical replicates, for each condition) were processed by MaxQuant software v.1.6.10.43 set on *Homo Sapiens* database (Uniprot taxonomy ID: 9606), against the Andromeda peptide search engine designed to analyze large datasets using a simple analysis workflow based on a probabilistic scoring model that produces normalized LFQ intensity values for each matched peptide. Trypsin was specified as proteolytic enzyme, cleaving after lysine and arginine except when followed by proline, and up to two missed cleavages were allowed along with the match between run option. The precursor ion tolerance was set to 5 ppm while the fragment tolerance was set to 0.5 Da. Cysteine carbamidomethylation of cysteine was defined as static modification, while oxidation of methionine and acetylation at the protein N-term were specified as dynamic modifications. LFQ analysis was statistically validated by applying a two-sample *t*-test of the LFQ intensities via Perseus software (v.1.6.10.43, Max Plank Institute of Biochemistry, Germany).

The protein network analysis related to those significantly modulated proteins was carried out by means of STRING tool (v.11.5) and of the Ingenuity Pathways Analysis (IPA) (QIAGEN Aarhus Prismet, Denmark) licensed software (S.6.3).

### 2.7 Molecular modeling studies

#### 2.7.1 Docking simulation

Molecular docking was performed by means of the software GOLD v. 5.8.1 [32] using the crystal structure of the BTB domain of Keap1 in complex with bardoxolone (PDB ID 4CXT) [33]. Water molecules were removed from the structure and hydrogens were added to the protein at physiological pH of 7.4 by means of H++ webserver [34]. Cys151 was simulated in its thiolate form as this represents the most predominant species at physiological pH. Indeed, Cys151 is surrounded by five basic residues which reduce its pK_a_ value favoring and stabilizing the thiolate form [35]. Protein structure was optimized as described elsewhere [36]. Ligands' structures were built by means of VEGA ZZ suite [37] and their conformational behavior was explored by following a Monte Carlo procedure as implemented in VEGA ZZ. The lowest energy conformations were selected and further optimized by DFT calculations at b3lyp/6–31+g(d,p) level of theory using Gaussian 16 [38]. The so-prepared ligands were then docked in the BTB domain of Keap1 as follows. The binding site was defined in order to contain all the residues within 10 Å from the native ligand and the ten poses were generated. The “allow early termination option” was deactivated and CHEMPLP was chosen as scoring function. The highest scored pose was selected for the analysis and representation. The binding free energy of the resulting minimized docking complexes was estimated by MM-GBSA method implemented in Amber18 [39] as described elsewhere [40].

#### 2.7.2 QM calculations

Quantum chemical calculation was performed by Gaussian 16, considering a simplified model reaction described elsewhere [41]. Cys151, VL, and Arg135 were modeled respectively as ethyl mercaptan, ortho-quinone and ammonium ion used as probe positive charge. For comparison purpose, the calculations were carried out on the same model without probe as well.

Reagents and adducts product in the keto form, at both C5 and C6 position, were optimized at wb97xd/6–311+g(d,p) in water using the CPCM (conductor like polarizable continuous model) implicit model solvent. This functional was extensively studied to model Michael-thiol addition and can take into account in well manner the long-way interaction like ion-dipole interaction and dipole-dipole interaction [42].

The frequency was also calculated to verify that the structure was in the energy minimum.

The ammonium ion was placed at a distance of 1.8 Å as seen for the best docking pose of the oxidized γV. Moreover, the five and six positions here considered of o-quinone are based on nomenclature for the same compound with substitution at C4 reported in Yang et al. [43].

The energy profile of the reaction was computed using the following equation [44]:

$$E_{\text{react}}=E_{\text{complex}}\;–\;\left(E_{\text{ion}}+E_{\text{reagents}}\right)$$

where E_react_ is the energy reaction, E_complex_ is the total energy of the keto adducts after the optimization, E_ion_ is the energy of ammonium ion, if present, and E_reagents_ is the sum of the individual energy of each reagent.

Due to the presence of ion-dipole interaction, the E_react_ was corrected with counterpoise method (SP) by Boys and Bernardi [45] performing a single point energy calculation on the same optimized adducts structures.

## 3 Results

### 3.1 Relevance of VL in the gut microbiota catabolism of GSE through *in vitro* fermentation

Several sets of data [15,23,46,47] converge to the notion that 5-(3′,4′-dihydroxyphenyl)-γ-valerolactone (VL) is a major component produced by the gut microbiota from flavan-3-ols [48].

The efficiency of the conversion of different precursors into VL varies across different degrees of polymerization suggesting a slower efficiency of conversion as degree of polymerization is higher. This observation aligns with the intrinsic requirements of the metabolic pathways leading to VL [46,49].

To provide clarity, this paper aimed to refine the understanding of the VL yield capacity of the GSE that is the subject of our present study. For this purpose, a well-established, and previously defined, technology was adopted to analyze the outcomes of fecal fermentation within different GSE fractions.

GSE was fractionated into five distinct fractions denoted as F1 through F5, using gel permeation chromatography [20].

These fractions represented 5.5 %, 2.9 %, 9.4 %, 21.6 %, and 48.8 % of the total GSE by weight, respectively (S.1-Table 2).

Subsequently, each fraction was characterized using both gel permeation chromatography (GPC) and mass spectrometry (MS), as illustrated in S.1.2 and S.1.3, respectively.

Briefly, the primary constituents of each fraction can be summarized as follows:

F1: No unmodified procyanidins detected.

F2: (Epi)catechin glycosides.

F3: Predominantly (epi)catechin

F4: Enriched in dimeric, trimeric, and tetrameric procyanidins, in descending order.

F5: Mainly composed of trimeric procyanidins, followed by dimeric and tetrameric and lower amount of larger polymers.

Each fraction was subjected to *in vitro* fermentation using a fecal sample as a source of gut microbiota (as described in S.2.1), and 75 μM of the average molecular weight (principal component) of the fraction, to standardize the conditions for the metabolic conversion. The analysis was carried out at T0 (0 min), T5 (5 h), and T24 (24 h).

LC-MS analysis relied on established bioconversion patterns for flavan-3-ols [14,49,50] and drew upon prior research findings [21,23,51,52], as detailed in S.2.- S1–S7.

As expected, present results confirmed VL as the predominant catabolite generated from the microbial metabolism of flavan-3-ols. While just traces of VL were detected in F1, for the remaining fractions, it accounted for the largest part of the overall pool of quantified microbial catabolites in all fractions.

To better gauge the significance of each fraction in the actual VL production from the GSE under scrutiny, the analytical data (reported in Supplementary data) were recalculated in terms of the actual mass conversion of GSE in VL at different times in different fractions (Table 1).

**Table 1:** Concentration (μM) of 5-(3′,4′-dihydroxyphenyl)-γ-valerolactone (VL) after 5 and 24 h of fermentation (T5 and T24) of 5 Ecovitis® fractions (F1, F2, F3, F4, F5). Incubation of fractions F1–F5 was carried out at constant concentration of 75 μM of the principal component in the fraction, as described in SI (S1.2.2). VL production has been measured at different fermentation times (T_5_, T_24_). Data are reported as mean ± standard deviation. From these values the normalized yield of VL expressed as mg for 100 mg of GSE in each fraction has been calculated.

Fraction	mg for 100 mg of GSE	MW (median)	Incubation mg/L	VL production μM/5hr	VL production μM/24hr	VL 5 h mg/100 mg GSE	VL 24 h mg/100 mg GSE
F1	5.5	456	34.2	0.08 ± 0.0	0.0 ± 0.0	0.003	0.000
F2	2.9	332	24.9	5.12 ± 0.04	9.38 ± 0.29	0.124	0.227
F3	9.4	290	21.8	55.93 ± 3.76	45.97 ± 0.11	5.028	4.132
F4	21.6	1154	86.6	60.41 ± 12.4	55.74 ± 2.60	3.136	2.893
F5	48.8	2018	151.4	10.29 ± 0.96	25.59 ± 7.66	0.690	1.716
Total						**8.98**	**8.97**

In summary: *i*) at T0 there was no VL detectable in the samples; *ii*) the maximal conversion was measured in F3 and F4 at T5 followed by a decrease of the yield due to further metabolism; *iii*) the production in F2 and F5, although slower, kept increasing at T24.

The observed slower but prolonged production of VL at high molecular weight PACs in F5 was likely due to steric hindrance slowing down the microbial processing, whereas in F2, it may be attributed to the presence of glycosylated (epi)catechins. These glycosylated compounds may require hydrolysis to release the aglyconic form, which would be further accessible to the gut microbiota, a phenomenon observed with other glycosylated flavonoids like anthocyanins [15].

### 3.2 Bioavailability of GSE metabolites

Building upon *in vitro* fermentation studies that identified VL as the primary procyanidin catabolite originating from GSE upon colonic microbiota catabolism, a targeted HPLC-ESI-MS/MS procedure was devised to provide evidence of bioavailability in healthy volunteers. The adopted procedure, indeed, aimed to quantify VL and its phase II metabolites (sulfate and glucuronide) within the urine of subjects treated with a low-(poly)phenol diet plus either GSE (2 capsules/day, 150mg/capsule) or placebo. Comprehensive baseline characteristics and details about the study population are presented in S.3.1 and S.3.2.

Illustrated in Fig. 1 are the excreted VL phase II metabolites: 5-(hydroxyphenyl)-γ-valerolactone-sulfate (co-elution of 3′- and 4′-sulfate) (Panel A) and 5-(hydroxyphenyl)-γ-valerolactone-glucuronide (Panel B) in urine samples of treated subjects. Notably, no free VL was detectable in urines of both GSE and placebo groups throughout the observation period. The quantitative assessment revealed that individuals receiving GSE had a urinary excretion of 20.8 ± 7.2 μmoles of sulfate conjugates (Panel A) and 16.0 ± 5.0 μmoles of glucuronide conjugate (Panel B) within a 24-h timeframe (results are expressed as mean ± s.e.m.). In the control, placebo treated group, the detected levels were: 3.7 ± 1.1 μmoles and 4.0 ± 1.0 μmoles for sulfate and glucuronide, respectively. The basal levels of VL detected in the placebo groups referred to the foods consumed by the participants during the (poly)phenol-low diet. Despite being scattered across a wide range of values, the data confirm and validate the notion of the bioavailability of VL. This is noteworthy despite the substantial inter-individual variability, as previously observed in a similar study on cranberry [53] and apple procyanidins and referred to individual diversity of microbiota [54].

In both *in vitro* and *in vivo* metabolic profiling, a coherent narrative unfolds detailing the conversion of procyanidins of GSE catalyzed by colonic microbiota enzymes, yielding a predominant bioavailable entity. This metabolic cascade unequivocally culminates in the emergence of VL, which, as expected, engages in a series of conjugative reactions eventually leading to phase II urinary metabolites. Assuming the existence of modest yet consistent VL plasma concentration throughout various phases, encompassing absorption, conjugation, excretion, and seemingly oxidation, this molecular entity is poised to play a noteworthy role in the health-enhancing effects attributed to GSE.

### 3.3 Nrf2 activation by VL: modulating regulatory feedback of inflammation

Considering intervention studies using (poly)phenols that consistently indicate a health-protective effect through their anti-inflammatory properties linked to their impact on cellular redox status and considering VL as the major bioavailable metabolite of GSE, we specifically addressed its relevance on the redox control of inflammation. Cells in various tissues face challenges that activate inflammation, the defense response that switches on the formation of different forms of oxidants. The impact of cellular redox status on inflammation is finely tuned by transcription factors NFκB and Nrf2, which interact through biochemical pathways involving electrophiles and nucleophiles. Nrf2 absence heightens NF-κB activity, while NFκB can modulate Nrf2's transcription, influencing target gene expression. This interplay is crucial as NFκB governs inflammation, which generates a kind of oxidative stress, while Nrf2 acts as an electrophile-activated counterbalance, aiming to repristinate the redox steady-state known as the “*golden mean*” [55].

We first confirmed using different analytical approaches on different cell lines what emerges as the most basic issue of the effect of VL as a suitable Nrf2 activator and NFκB inhibitor. Using TNFα as challenge operating an activation of inflammation associated to a shift towards a more oxidizing cellular environment, we observed upon addition of VL:

a) on HEK293 cells with the cell reporter the Nrf2 activation;
b) on R3/1 cell with the cell reporter the decrease of NFκB;
c) on Caco-2 cells the decrease of NFκB and its activated phosphorylated form.

**Fig. 2:**
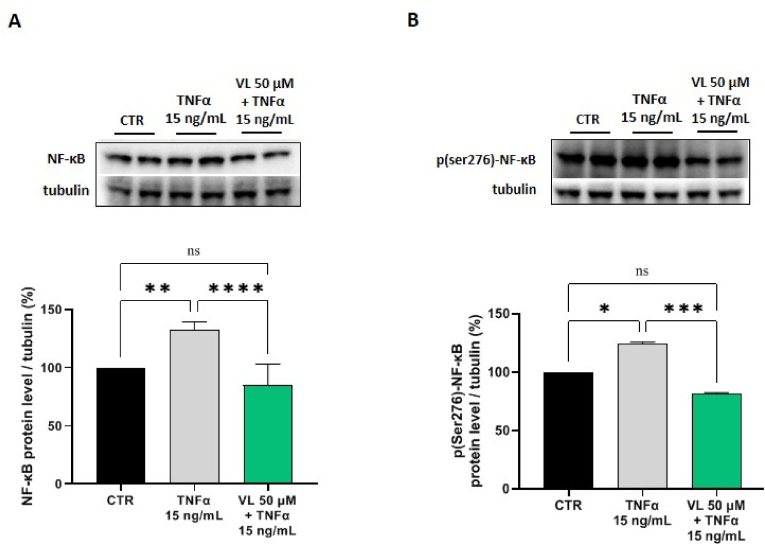
Effect of VL on NFkB (A) and p(ser276)-NFkB (B) protein levels in human intestinal cells. Notably, Caco-2 cells were treated with VL at the concentration of 50 μM for 24 h. Subsequently, its ability to modulate the NFkB (A) and p(ser276)-NFkB (B) protein levels was assessed by Western blotting. The data points represent the averages ± s.d. of four independent experiments in duplicate. All data sets were analyzed by one-way ANOVA followed by Tukey's post-hoc test. C: control sample; ns: not significant; (*) p < 0.05, (**) p < 0.01, (***) p < 0.001 and (****) p < 0.0001.

In more detail, VL dose-dependently (50–150 μM) acted as promotor of the Nrf2 nuclear translocation in HEK293 cells (S.5-Fig. 1) and inhibited in a dose range 10–100 μM the NF-κB signaling pathway (S.5-Fig. 2).

As shown in Fig. 2, after treating Caco-2 cells with TNFα (15 ng/mL), the NFκB protein levels increased up to 132.2 ± 7.3 % (p < 0.01). The pretreatment of Caco-2 cells with VL (50 μM) reduced the TNFα-induced NFκB protein by 15.09 ± 18 %, versus control cells (Fig. 2A). In parallel, the effects of VL on the active p(ser276) NFκB which is able to translocate in the cellular nuclei, were evaluated, demonstrating that, after TNFα treatments (15 ng/mL), the p(ser276)-NFκB protein increased up to 124.1 ± 1.7 % in Caco-2 cells (p < 0.05). The pretreatment of Caco-2 cells with VL (50 μM) reduced the TNFα-induced p(ser276)–NF–κB protein levels by 18.49 ± 1.1 %, versus control cells (Fig. 2B). The pre-treatments of human Caco-2 cells with VL successfully rescued the TNFα induced inflammatory status. Indeed, no significant differences were observed between the NFκB protein levels after pre-treatments with VL versus the control, untreated samples (Fig. 2A). On the contrary, VL reduces the active p(ser276)-NFκB protein levels below the control condition (Fig. 2B).

By extrapolating the amount of excreted VL, we can assume that within 24 h the dose of 300 mg of GSE, VL reaches the colonocytes at a concentration of 2.52 nmol cm^2^, a value which is of the same order of magnitude in respect to that able to exert a robust and significant anti-inflammatory and antioxidant activity as found in vitro experiments (Caco-2 cells, 4.6 nmoles per cm^2^ per 24 h). More details on this aspect are described in Supplementary Information (S.5.2.3).

### 3.4 VL interaction with Keap1: computational evidence supporting Nrf2 activation

Phenolic antioxidants, despite their free radical scavenging capacity, activate Nrf2 operating as electrophiles [56]. This apparent paradox is resolved by realizing that these antioxidants are prone to oxidation to the quinone form, in turn competent for the activation of Nrf2 as electrophile. This concept changes how we view phenolic antioxidants, seeing them not just as free radical scavengers but as contributors to the redox feedback loop that supports cellular redox steady-state required to maintain homeostasis when challenged by pro-inflammatory stressors [18,55]. Considering this array of information, we turned to seek a deeper understanding of the interaction between VL and Keap1, the main controller of Nrf2 activation.

Molecular docking was carried out to probe the binding mechanism of VL to Keap1. Catechols are very easily converted to quinones by an oxidative transition catalyzed by virtually any oxidative enzyme, metal ions [57] and oxidants [58,59]. It can therefore be assumed that under conditions of oxidative/inflammatory stress, when a series of reactive species are generated, usually referred to as ROS, the ortho-diphenol moiety of VL undergoes a redox transition to its reactive quinone form. Notably, dioxygen itself facilitates this redox transition within cells. It has been observed, indeed, that the ability of different phenolic compounds to activate Nrf2 decreases significantly dropping from the commonly used 20 % level used in cell experiments to just 2 % [60].

Therefore, keeping for granted the easy redox transition from catechol moiety to quinone our analysis was addressed on both VL and its oxidized form focusing on the BTB domain of KEAP1 as it contains Cys151 proved to be the most reactive towards electrophilic species [61]. The highest reactivity of Cys151 is attributed to the presence of five basic residues (*i.e.* His129, Lys131, Arg135, Lys150 and His154) in its close proximity which lower the pK_a_ of its thiol group shifting the equilibrium towards the thiolate form at physiological pH [35].

**Fig. 3:**
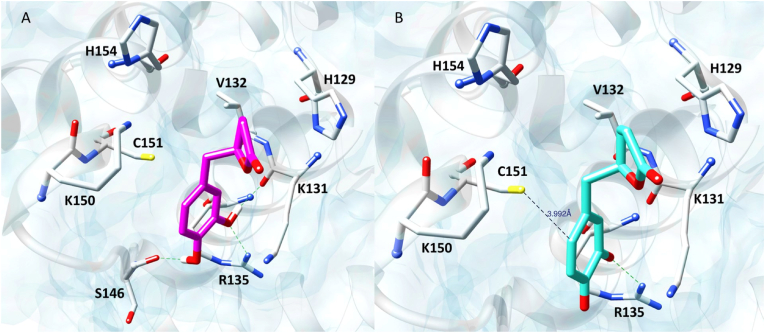
Panel A. Docking pose of VL (magenta sticks) within Keap1 binding site. Panel B. Binding mode of the oxidized VL (cyan sticks). Green dashed lines represent H-bond interactions. The binding free energy (ΔG) values obtained for VL and its oxidized form are −17.03 kcal/mol and −16.98 kcal/mol, respectively.

The docking results revealed that both entities can assume similar binding modes within Keap1 binding site (Fig. 3), with the respective catechol and quinone rings stabilized by a π-sulfur interaction with Cys151 and a H-bond with Arg135. Concerning VL, the catechol moiety enables the formation of additional H-bonds involving the backbone carbonyl groups of Ser146 and Lys131 (Fig. 3A). Instead, the VL moiety is involved in p-p stacking with His129 plus hydrophobic contacts with Lys131 and Val132. Due to these contacts, also the valerolactone moiety, besides the catechol, might play a role in orienting the ligand in the binding site favoring the exposure of the electrophilic warhead to the thiolate group of Cys151. Indeed, docking simulations involving the sole catechol ring revealed that it is unable to assume a pose conducive to the reaction with Cys151 (results not shown), thus indicating the pivotal role of the lactone ring in the VL recognition and binding.

**Fig. 4:**
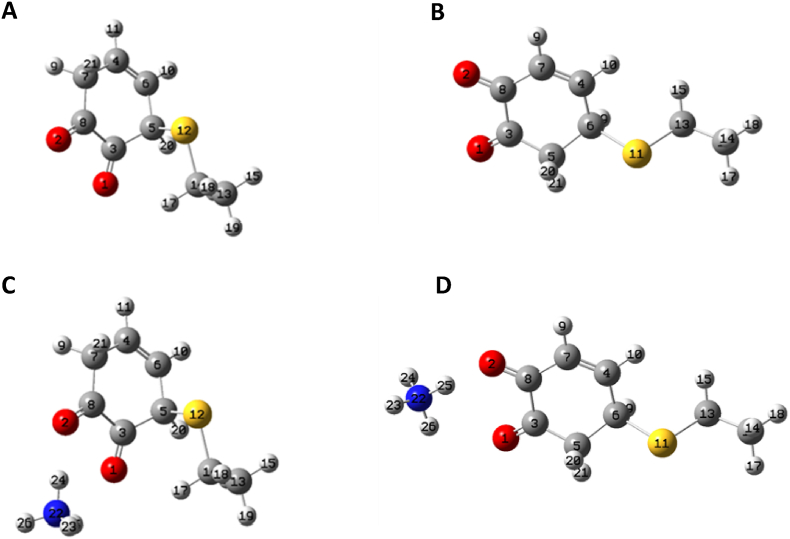
Gaussian optimized structures of 5′-adduct and 6′-adduct without ammonium ion (panel a and b) and structures of 5′-adduct and 6′-adduct with NH4+ (panel C and D).

Interestingly, in the oxidized form the reactive quinone moiety might assume a suitable orientation for the reaction with Cys151 adopting a distance between the thiol group and the electrophilic sp2 C at position 6′ of 3.992 Å (Fig. 3B). The binding mode obtained for the oxidized VL suggested that Arg135 could polarize the ligand, increasing its electrophilicity, thus further promoting the nucleophilic attack of Cys151 towards the carbon atom at 6′ position. On this basis, QM calculations were carried out to confirm the position of the quinone ring involved in Michael addition, by evaluating the reaction energy of the two potential adducts of the nucleophilic attack at 6’ (1,4-Michael addition) and 5’ (1,6-Michael addition) positions [62], with and without the ammonium ion used as probe to simulate the positive charge of Arg135 (Fig. 4).

The reaction energy was calculated by the Density Functional Theory (DFT) which allows to analyze the stereo-electronic properties of the simulated systems. The computed energy values showed that all the simulated adducts at 5′ and 6′ positions are stable as indicated by the negative energy values. Interestingly, the presence of a positive charge further stabilizes the adducts of Michael addition lowering the energy of the reaction. In detail, when the reaction involved the 5′ position of the quinone, the energy value decreased from −11.64 kcal/mol to −23.47 kcal/mol in the presence of the positive charge probe. The same trend was observed for the reaction at 6′ with a reduction of the energy from −18.63 kcal/mol to −26.76 kcal/mol. Overall, the outcomes confirmed the favourable role of the interaction with Arg135 and revealed that the adduct at the 6’ position is the most stable further supporting the binding mode yielded by docking simulations.

### 3.5 Proteomic studies

After accumulating substantial evidence regarding the bioavailability of VL produced by microbiota and the effects of the dynamic interplay between NFκB and Nrf2, we opted to further enrich our understanding through the utilization in Caco-2 cells of label-free LC-MS quantitative profiling (LFQ analysis). This robust quantitative proteomic methodology, indeed, furnishes insights into the relative protein contents across two or more biological samples [63].

The subsequent sets of experimental conditions, focusing on the regulation of TNFα activation, were subjected to comparison: i) TNFα vs. CTR; ii) VL vs. CTR; iii) TNFα-VL vs. TNFα. This comparative analysis aimed to decipher the intricate molecular pathways implicated in the initiation and progression of the process.

In total, 3828 proteins were successfully identified and quantified. The modifications of protein expression that attained statistical significance are depicted in S.6-Fig. 2. The robustness of the biological and technical replicates was established by computing Pearson's linear correlation coefficient, which surpassed 0.98 for all the paired experimental conditions (data not shown).

#### 3.5.1 TNFα vs. CTR

**Fig. 5:**
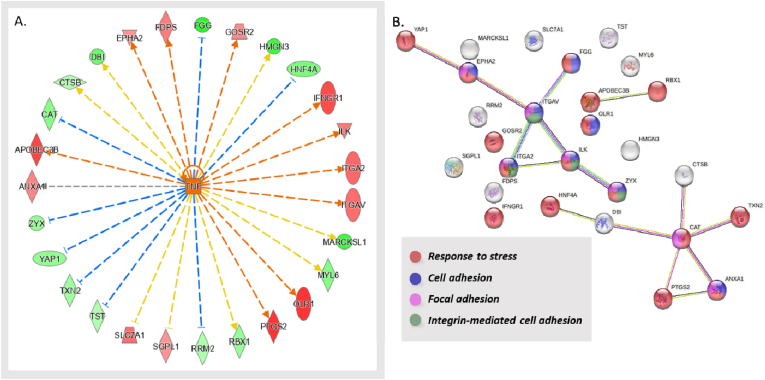
Panel A. IPA analysis focusing on the onset and development of the inflammatory state (TNFα vs. CTR) identified TNFα as a potential upstream regulator; the wheel of modulated proteins is consistent with TNFα stimulation. *Color legend:* red represents the upregulated genes, green the downregulated. The intensity of the color is related to the intensity of up- or down-regulation. The orange line leads to activation and a blue line leads to inactivation. The yellow line indicates findings that are not consistent with the proteomics results obtained. Panel B. STRING analysis of the modulated gene products determined by IPA, shown as a protein–protein interaction network obtained; the color/s of each node (gene product) reflect/s the functional enrichment analysis performed whose code is shown below the network.

The compilation of differentially expressed proteins upon TNFα exposure in contrast to the control group is presented in Fig. 5. IPA data analysis discloses the differential protein expression pattern consistent with the phenotypic inflammatory condition. Among the 27 proteins implicated in the pro-inflammatory stimulus, a subset of 9 proteins exhibited clustering within the integrin-mediated cell adhesion pathway, and, more broadly, within focal adhesion (Fig. 5). This clustering is of particular significance given the crucial role that cellular adhesion to other cells or the extracellular matrix plays in various processes, including inflammation.

**Fig. 6:**
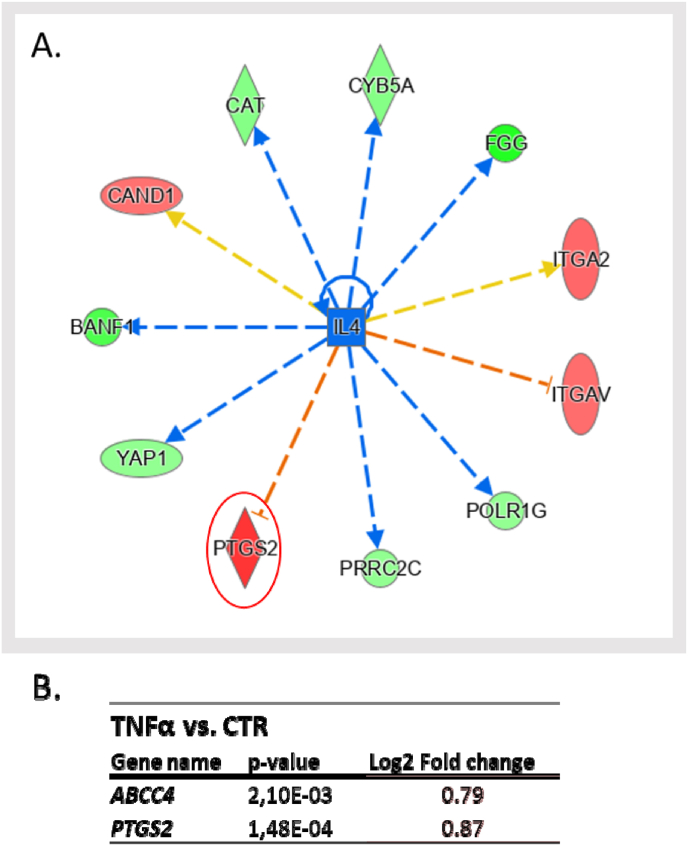
Panel A. IL4 was enriched as a potential upstream regulator determined in the TNFα vs. CTR condition by means of IPA analysis (LFQ); the dataset allows a hypothesis of IL4 inhibition to be generated. PTGS2 is circled in red due to the high interest as to the involvement of this protein, whose Log2 fold-change and p-value values are shown in panel B, alongside the Log2 fold-change and p-value values of ABCC4 closely related to PTGS2. *Color legend:* red represents the upregulated genes, green the downregulated. The intensity of the color is related to the intensity of up- or down-regulation. The orange line leads to activation and a blue line leads to inactivation. The yellow line indicates findings that are not consistent with the proteomics results obtained.

Within the cascade of findings, also two gene products linked to the prostaglandin pathway emerged as upregulated by TNFα (Fig. 6). The first, prostaglandin-endoperoxide synthase 2 (PTGS2), also known as COX-2 (cyclooxygenase 2), is an inducible enzyme that becomes overexpressed in inflammatory states [64]. The second notable protein is ABCC4 (ATP-binding cassette sub-family C member 4), a transporter intricately involved in the conveyance of prostaglandin E2, a principal product generated by the COX-2 enzyme [65].

In accordance with the transition to an inflamed state we detected that the upregulation of pro-inflammatory proteins coincides with the downregulation of the anti-inflammatory interleukin IL-4 (Fig. 6).

Additionally, a salient indication apparent in TNFα-treated cells involves an altered mitochondrial function, which indirectly hints at a potential reduction in cellular energetics (S.6- Fig. 3). The expression of several proteins supporting mitochondrial functions is distinctly diminished upon exposure to TNFα. Particularly noteworthy is the significant decline observed in the constituents responsible for the ultimate steps of the electron transport chain and oxidative phosphorylation process, namely COX5A, COX5B, ATP5D, ATP5I, and ATP6V1G1.

#### 3.5.2 VL vs CTR

As reported in Table 2, VL overexpressed a set of proteins involved in the mitochondrial oxidative phosphorylation and as expected, the proteins embracing the global gene expression shift operated by Nrf2, namely antioxidant response element (ARE)-mediated genes such as CAT, HMOX and TXN.

**Table 2:**
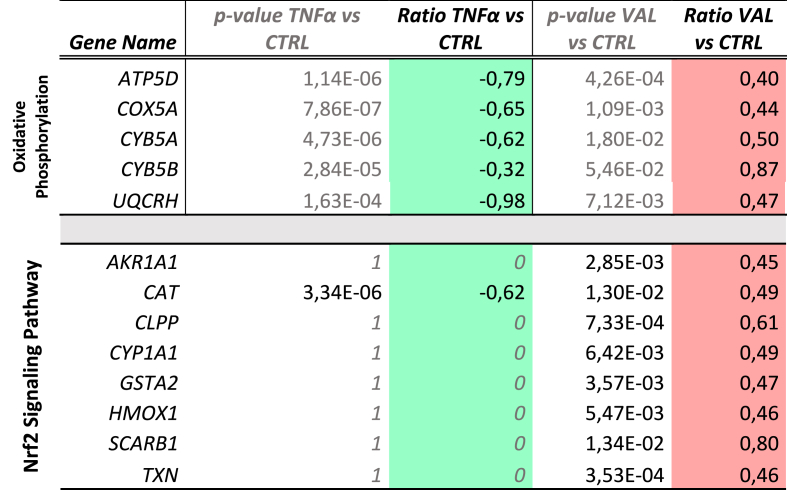
Overview of the gene products modulated by VAL treatment under physiological conditions sorted according to their involvement in the oxidative phosphorylation pathway and the Nrf2 signaling pathway; p-value and log2 fold-change values calculated under the experimental conditions TNFα vs. CTR and VAL vs. CTR are given for each gene product to emphasize the trend inversion. The conditional formatting used is based on a color code whereby green indicates the lower value and red the higher value.

#### 3.5.3 TNFα-VL vs TNFα

From a global standpoint, upon comparing the differential expression of proteins between TNFα vs CTR and TNFα vs TNFα-VL, a clear pattern emerges, indicating that the proinflammatory shift in gene expression induced by TNFα is nearly entirely counteracted by VL. This pronounced reversal of the trend is particularly highlighted in the case of PTGS2 and the anti-inflammatory IL-4 pathway (S.6-Fig. 4).

**Fig. 7:**
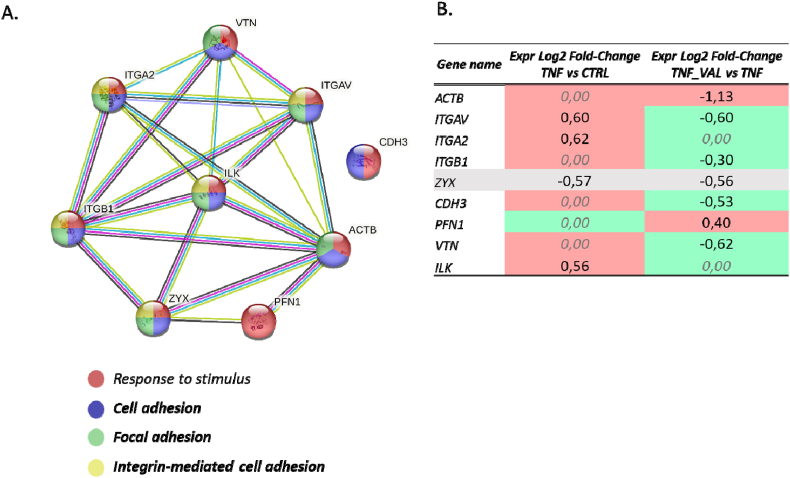
**Panel A.** STRING analysis of the modulated gene products in the condition TNFα-VAL vs TNFα mainly involved in the cell adhesion, showed as a protein–protein interaction network; the color/s of each node (gene product) reflect/s the functional enrichment analysis performed and the legend is shown below the network. **Panel B.** The p-value and log2 fold-change values calculated under the experimental conditions TNFα vs. CTR and TNFα-VAL vs. TNFα are given for each gene product to emphasize the trend inversion. The conditional formatting used is based on a color code whereby green indicates the lower value and red the higher value.

This opposite trajectory is strikingly evident also for the integrin proteins associated with focal adhesion (Fig. 7). Reported data encapsulate the log2 fold change of integrin proteins within the two experimental groups, alongside a protein-protein interaction network that underscores their shared involvement in the pertinent signaling pathway.

Except for the expression values of zyxin, which has a regulatory rather than effector role, the entire panel of enriched proteins in this context showed a reversed trend among the two experimental conditions.

**Fig. 8:**
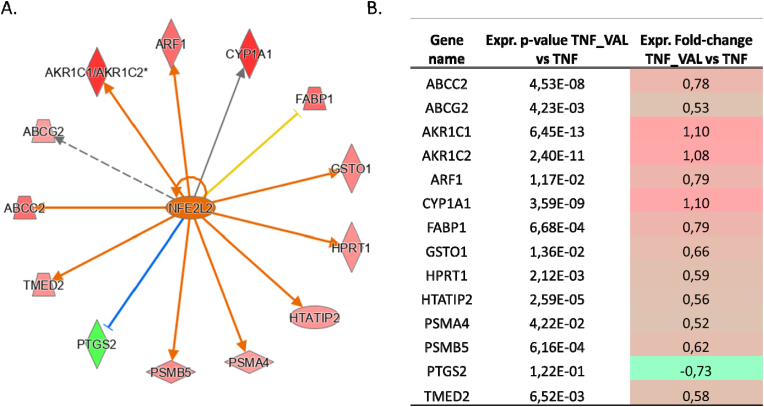
**Panel A** depicts the differentially regulated proteins in the analysis consistent with Nrf2 activation (IPA analysis). **Panel B.** Overview of the p-value and log2 fold-change values calculated under the experimental conditions TNFα-VAL vs. TNFα given for each gene product to emphasize their up-regulation extent, except for the consistent down-regulation of PTGS2.

In summary as previously reported, VL exhibited significant modulation of 13 gene products pertinent to Nrf2 activation in control cells, with this effect becoming even more pronounced when confronted with the challenge brought by TNFα. The comparison between TNFα_VL and TNFα scenarios reveals the presence of Nrf2 among the putative upstream regulators. This enriched network described by the log2 fold-change values plot, distinctly indicates a pronounced activation of the signaling pathway by VL, even in the presence of TNFα challenge (Fig. 8).

Lastly, considering oxidative phosphorylation, the TNFα induced downregulation of proteins inherent to the electron transport chain in mitochondria was only incompletely reverted by VL.

## 4 Conclusions on the perspective of the para-hormetic effect of GSE

The influence of nutrition on civilization, quality of life, and health maintenance of humans can be categorized into three significant epochs. First, the domestication of animals and vegetables, more than 10,000 years ago, enhancing energy availability. Second, the discoveries made in the last century regarding essential elements necessary for preventing deficiency diseases. Finally, in recent decades, the recognition of fruits and vegetables as sources of xenobiotic phytochemicals capable of bolstering resilience against potential disruptors of homeostasis, which could manifest as diseases.

These compounds, and thus the plant-based dietary sources that contain them, challenge easy classification as either essential nutrients or distinct botanical drugs. They often confer a diverse array of health benefits, often referred to as a “Panacea,” reminiscent of the Goddess of universal remedies. The observed effect is addressed to tightening the evolution of inflammatory diseases [66]. Among these phytochemicals are included (poly)phenols [67] or glucosinolates [68]. This perspective unveils the intricate ecological and nutritional interplay between plants and omnivores, aptly termed the “Labyrinth of Omnivore” [69].

This modern scientific approach frequently aligns with traditional practices and wisdom in nutrition. While these compounds might not have direct links to precise pharmacological targets or deficiency syndromes resulting from their dietary absence, they are recognized as pivotal agents that aid in maintaining fluctuations around the optimal homeostatic state crucial for overall well-being [55] named “Golden Mean” from Aristotelian Nichomachean Ethics book.

In this study, we combined original analytical and experimental evidence with a discussion on foundational information concerning the broader concept of nutritional hormesis [70,71].

The concept of hormesis suggests that Nrf2, acting as a redox-sensitive transcription factor, triggers a natural defense system against stressors. It can be activated by different stress-inducing factors like phytochemicals, exercise or radiation [72].

Nrf2 works in tandem with other transcription factors to reinforce adaptive strategies, thereby boosting resilience [73]. This mechanism drives hormetic dose responses, protecting biological systems and conserving resources through its controlled reaction. Nrf2's function as a hormetic mediator offers valuable insights into overall biological resilience and susceptibility to diseases [72] in the broader context of the health biology [71,74].

The closer concept of “Para-hormesis” [18] has been proposed in relation to antioxidant phytochemicals found in vegetables. They enhance the body's resilience without posing the challenge of inducing toxicity as a genuine hormetic agonist. This concept is closely intertwined with the cellular redox status. Shifting towards a more oxidized environment serves as the response to a challenge, while reverting back to a more nucleophilic environment signifies the restoration of a healthy homeostatic balance, preventing pathology descending from excessive inflammation [55].

At the heart of these homeostatic processes lies their distinctive biochemistry. The pioneering work of Albert Szent-Györgyi during the 1930s [75] laid the foundation for speculation surrounding the health-promoting potential of (poly)phenols. Much of this speculation is rooted in their redox properties.

The current roadmap outlining the health effects of (poly)phenols is structured upon three key elements, bridging redox-chemistry with control of inflammation.

1 Within cells, diverse challenges stimulate an upsurge in oxidants, assuming roles as activators of inflammation as the basic defense mechanism [76,77].
2 Poorly controlled inflammation in terms of space and time has the potential to inflict damage unless effectively counterbalanced through the controlled feedback loop operated by electrophiles produced during inflammation [19,55].
3 Phytochemicals, including antioxidant (poly)phenols, contribute to this feedback loop evolving into electrophiles minimizing the oscillations of the steady-state of redox state recognized as a cornerstone of health [18].

The current experimental study, coupled with a short survey of the scientific evidence bolstering the concept of nutritional hormesis, was designed to unravel the intricate biochemical pathways that form the foundation for the observed protective health benefits associated with grape seed (poly)phenols within the framework of the para-hormetic effect.

Employing GSE, we substantiated the concept that the bioavailable VL primarily stems from microbiota-driven processes. Moreover, through the strategic fractionation of the GSE into discrete segments characterized by varying polymerization levels, our findings consistently linked elevated polymerization degrees to a more gradual release profile of VL. This deliberate and controlled release mechanism holds the potential to enhance the bioefficiency of VL, thus enabling prolonged and consistent patterns of absorption. These distinctive characteristics underscore VL, derived from lengthier polymer structures, as a compelling candidate responsible for augmenting the observed health benefits attributed to GSE.

The bioavailability of VL was validated by detecting urinary metabolites, namely sulfate and glucuronide derivatives. Notably, considerable variability was observed among different subjects, a phenomenon in agreement with findings from earlier studies on apple procyanidins [53,78,79]. This variability can be reasonably enough linked to the diverse nature of individual microbiota.

It is foreseeable that the para-hormetic response induced by GSE we observed is influenced by various external and individual factors that affect the formation of VL in gut microbiota, as well as its systemic absorption and metabolism. It has been reported that the efficiency of gut microbiota in producing VL shows interindividual variability, attributed to factors such as diet, age, and metabolic disorders [[80], [81], [82]]. To better comprehend and rationalize this variability, the concept of metabotypes has been introduced, classifying individuals into groups with similar metabolic capacities [83]. Additionally, external factors, such as the food matrix in which polyphenols, including GSE, are consumed, can impact the efficiency of GSE catabolism [84]. Host gene polymorphisms are also expected to influence VL metabolism. For instance, gene polymorphisms of enzymes like UGT and sulfotransferases, crucial for the conjugation of VL in phase II metabolism, differ among individuals [85].

In summary, given the expected diversity in individual responses falling within a certain range following the intake of any food, xenobiotic, or drug, it would be surprising if this were not true for GSE as well. Nonetheless, this is a possibly relevant issue that deserves future specific studies addressing population.

The presence of conjugated derivatives of VL in urine may serve as indirect evidence of the presence of a low steady-state concentration of the native compound in human fluids, which could also be derived from intracellular metabolism [86]. On the other hand, the biological effect of VL on our cellular model of control of inflammation was clear and fully consistent with previous observations.

This discrepancy requires a discussion aiming to pinpoint the actual species derived from VL competent for the biological effects. Again, a sustainable rationale emerges from the peculiar chemistry of VL and specifically its catechol moiety.

It is important to highlight that, among VL and its phase II metabolites, only VL possesses an ortho-diphenol moiety capable of transitioning into the electrophilic quinoid form, rendering it competent for the generation of Michael adducts with KEAP1 thiols. Therefore, in principle, phase II metabolites of VL can be regarded as circulating inactive metabolites suitable to enzymatic reversion to the bioactive form. The process of de-glucuronidation of monoglucuronide flavonoids has been observed during inflammation, facilitated by beta-glucuronidase released from stimulated neutrophils [87,88]. Additionally, the activity of beta-glucuronidase is pH-dependent and is enhanced by the increased production of lactate due mitochondrial dysfunction [89]. Sulfate metabolites, such as resveratrol sulfates [90], may also undergo hydrolysis in tissue samples catalyzed by members of the sulfatase family, leading to the regeneration of the parent compound.

In summary while the enzymatic conversion of phase II metabolites to the bioactive parent compound emerges as necessary to explain the systemic activity of GSE, more specific investigations are warranted to fully elucidate details of this intricate process.

The kinetics and thermodynamics governing the redox transitions via single hydrogen transfer within the catechol moiety of phenolic compounds strongly favor the transition towards quinone formation [78]. This chemical transition, deeply characterized *in vitro*, contributes to a marked free radical scavenging activity, a feature extensively interpreted as a noteworthy antioxidant effect.

On the other hand, intertwined with this concept is the rapid formation of an electrophilic quinone, competent for exert cytotoxic or cell signaling effects [91,92]. Indeed, the interaction between quinones and nucleophilic sites within proteins has the potential to modify their functionality [92]. Protein thiols exhibiting low pKa values, which render them particularly adept electrophiles, might serve as sensors capable of perceiving an oxidizing environment. This, in turn, triggers a feedback signal that ultimately leads to the restoration of nucleophilic tone [55]. In summary, the effect of VL derived from GSE is the activation of a positive mild “oxidative stress”.

This dualistic and seemingly paradoxical behavior of phenolic antioxidants was elegantly deciphered through the pioneering work of Cecil Pickett, who discovered the regulatory activation of cytoprotective genes by various electrophilic agents [93]. In the context of (poly)phenols, it was implicitly understood that the redox transition from nucleophilic to electrophilic states must precede the formation of Michael adducts on the Keap1 protein, a pivotal component of the cellular protective redox regulatory machinery. The comprehensive delineation of this pathway, focused on the modulation of Nrf2 activation, laid the groundwork for a paradigm shift in the perception of phenolic antioxidants. They transcend the role of mere free radical scavengers to emerge as hormetic regulators of the cellular redox status [94].

To delve further into the mechanism, we generated molecular docking data substantiating the Michael addition of oxidized VL to the pivotal regulatory Cys151 within Keap1. Additionally, we also observed the significance of the valerolactone moiety in accurately positioning VL within the cleft housing the regulatory Cys.

Collectively, this amalgamation of information compellingly implies that the discernible urinary metabolites of VL primarily consist of conjugates that have evaded redox transitions and interactions with nucleophiles.

In the latter segment of this study, we delved more extensively into the pattern of gene expression stemming from the interplay between the pro-oxidative signals prompted by TNFα stimulation and the anti-inflammatory impact of Nrf2 activation. This interplay appears intricately linked to an augmented nucleophilic milieu.

Through LC-MS quantitative profiling (LFQ analysis) of Caco-2 cells treated with VL and TNFα, robustly validate VL's anticipated role as an antioxidant and detoxifying agent with notable anti-inflammatory effects. Particularly noteworthy is VL's distinct efficacy in counteracting TNFα-induced shifts in gene expression, highlighted by the clustering of integrin-mediated cell adhesion and focal adhesion pathways.

Furthermore, VL demonstrates a discernible tendency to reverse the diminished expression of proteins associated with mitochondrial functions resulting from TNFα exposure. This evidence emphasizes the far-reaching impact of Nrf2 on upholding homeostasis, reaching into the realm of bioenergetics. This encompasses the well-established suppression of fat production, facilitation of fatty acid breakdown, and an all-encompassing promotion of energy-releasing catabolic processes in alignment with our proteomic insights into mitochondrial function.

In the realm of integrative medicine, a salient observation emerges: dynamic regulation of Nrf2 as a common denominator spanning a diverse spectrum of challenges. These encompass not only autoimmune disorders, respiratory afflictions, cardiovascular intricacies but also an array of cancer manifestations. The dataset at hand contributes mechanistic validation, thereby fortifying the prevailing body of evidence that situates GSE within the cohort of phenolic phytochemicals taking an active role in preserving the intricate steady state of redox homeostasis. This equilibrium, pivotal for overall well-being, can be aptly characterized as a continual, energy-demanding endeavor to uphold good health.

## Funding

LDV and GF are supported as Ph.D. student and temporary researchers (RTDA) by Ministero dell’Università e della Ricerca PON “Ricerca e Innovazione” 2014–2020, Azione IV.4—“Dottorati e contratti di ricerca su tematiche dell'innovazione” and Azione IV.6—“Contratti di ricerca su tematiche Green”. CUP G41B21009720002.

Distillerie Bonollo Umberto S.p.A. partially funded the project.

## Declaration of competing interest

The authors declare the following financial interests/personal relationships which may be considered as potential competing interests:

Giancarlo Aldini is PI of a research contract signed by the Università degli Studi di Milano with Distillerie Bonollo Umberto S.p.A.

Larissa Della Vedova is student of a Ph.D program co-sposnored by Distillerie Bonollo Umberto S.p.A.

Paolo Morazzoni has a consultancy agreement with Distillerie Bonollo Umberto S.p.A. in the field of research and development of new products based on the supply chain of Vitis vinifera.

Francesca Gado is a temporary researcher (RTDA) granted by Ministero dell’Università e della Ricerca PON “Ricerca e Innovazione” 2014–2020 with a research program shared with Distillerie Bonollo Umberto S.p.A.

Fulvio Ursini Emeritus Professor of Biochemistry, Scientific consultant.

## Data Availability

Data will be made available on request.
