# Supplementary material for: Unraveling the parahormetic mechanism underlying the health-protecting effects of grapeseed procyanidins
Source: Redox Biol. 2023 Dec 7;69:102981. doi: 10.1016/j.redox.2023.102981 (PMC10770607; doi:10.1016/j.redox.2023.102981)
Supplement: Multimedia component 1 [file mmc1.docx]

**S.0. *Chemicals and reagents***

Bile salts, soluble starch, (+)-arabinogalactan, tryptone, yeast extract, xylan from birchwood, L-cysteine, hydrochloride monohydrate, guar gum, inulin, Tween 80, buffered peptone water, Dulbecco’s phosphate buffer saline (PBS), casein sodium salt from bovine milk, pectin from citrus fruits, mucin from porcine stomach-type III, CaCl_2_, KCl, NaCl, NaHCO_3_, anhydrous K_2_HPO_4_, KH_2_PO_4_, MgSO_4_ monohydrate, FeSO_4_ heptahydrate, resazurin redox indicator, formic acid, (+)-catechin, (−)-epicatechin, phenylacetic acid, 4′-hydroxyphenylacetic acid, 3′-hydroxyphenylacetic acid, 3′,4′-dihydroxyphenylacetic acid, 3-phenylpropanoic acid, 3-(4′-hydroxyphenyl)propanoic acid, 3-(3′-hydroxyphenyl)propanoic acid, 3-(3′,4′-dihydroxyphenyl)propanoic acid, benzoic acid, 4-hydroxybenzoic acid, 3-hydroxybenzoic acid, 3,4-dihydroxybenzoic acid, 3,4,5-trihydroxybenzoic acid, benzene-1,2,3-triol, benzene-1,3,5-triol, 3,4-dihydroxybenzaldehyde, and 4-hydroxybenzaldehyde were purchased from Sigma-Aldrich (St Louis, MO, USA). 5-(4′-Hydroxyphenyl)-γ-valerolactone, 5-(3′-hydroxyphenyl)-γ-valerolactone, and 5-(3′,4′-dihydroxyphenyl)-γ-valerolactone were synthesized in house [26, 27]. 5-(3’,4’-dihydroxyphenyl)-γ-valerolactone D4 used as internal standard (IS) was synthetized and characterized by Prof. L Fumagalli (submitted paper).

Epigallocatechin-3-*O*-gallate (EGCG), dimer A2, dimer B2 and trimer BB were purchased from Extrasynthese (Genay Cedex, France). Tetramer BBB and pentamer BBBB were purchased from PlantaAnalytica (New Milford, CT, USA). All solvents and reagents were UHPLC-grade and were purchased from VWR International (Milan, Italy), unless otherwise indicated. Ultrapure water from MilliQ system (Millipore, Bedfort, MA, USA) was used throughout the experiment.

Ultrapure water was prepared by a Milli- Q purification system (Millipore, Bedford, MA, USA). Dulbecco's modified Eagle's medium (DMEM), fetal bovine serum (FBS), phosphate buffered saline (PBS), penicillin/streptomycin, chemiluminescent reagent, flasks and multi-well plates were purchased from Euroclone (Milan, Italy). Dimethyl sulfoxide (DMSO), bovine serum albumin (BSA), TNFα, RIPA buffer, the antibodies against tubulin, NF-κB and p(ser276)-NF-κB were bought from Sigma-Aldrich (St. Louis, MO, USA). Phenylmethanesulfonyl fluoride (PMSF), Na-orthovanadate inhibitors and the antibodies against rabbit Ig-horseradish peroxidase (HRP) and mouse Ig-HRP were purchased from Santa Cruz Biotechnology Inc. (Santa Cruz, CA, USA). The inhibitor cocktail Complete Midi from Roche (Basel, Swiss); Mini protean TGX pre-cast gel 7.5% and Mini nitrocellulose Transfer Packs from BioRad (Hercules, CA, USA).

Iodoacetamide (IAA), tris(2-carboxyethyl)phosphine (TCEP), tetraethylammonium bromide (TEAB), 3-(4,5-Dimethyl-2-thiazolyl)-2,5-diphenyl- 2H-tetrazolium bromide (MTT), IL-1α, sequencing-grade trypsin (Roche), formic acid (FA), trifluoroacetic acid (TFA), acetonitrile (ACN) and all ultra-pure-grade (99.5%) solvents used in LC-MS analysis were obtained from Merck KGaA, Darmstadt, Germany. SPE Hypersep C18 column (100 mg/mL) were from Thermo Scientific (Milan, Italy). S-TRAP^TM^ columns were provided by Protifi (Huntington, NY, USA).
