# Supplementary material for: Unraveling the parahormetic mechanism underlying the health-protecting effects of grapeseed procyanidins
Source: Redox Biol. 2023 Dec 7;69:102981. doi: 10.1016/j.redox.2023.102981 (PMC10770607; doi:10.1016/j.redox.2023.102981)
Supplement: Multimedia component 2 [file mmc2.docx]

**S.1. *Fractioning* of grape seed extract and fractions characterization by GPC (*gel permeation chromatography)*** **and LC-MS**

# S.1.1. Proanthocyanidins fractioning from grape seed extract

One gram of grape seed extract was dissolved in 31 mL of MeOH-water and formic acid (500:500:2) solution, and then separated into 5 fractions according to the method described by Kennedy *et al.* [20]. Briefly, 30 mL of the procyanidin solution (968 mg) was loaded onto a Sephadex LH-20 column (40x150 mm) equilibrated with MeOH-water and formic acid (500:500:2) and connected to a preparative chromatographic system (GE Healthcare). The column was eluted in 5 successive isocratic passes described in supplemental table S0. The pooled fractions were rotary evaporated, freeze- dried and stored at -22 °C

**Table 1** **-** Solvent systems and elution volumes used to fractionate proanthocyanidins. (a) Solvents applied sequentially from top to bottom with the balance within each solvent system consisting of water. Solvent–water mixtures contained 0.2% (v/v) formic acid.

| **Solvent system (a)** |  | | **Volume (mL)** |  |
| --- | --- | --- | --- | --- |
| 60% (v/v) methanol |  | | 200 |  |
| 75% (v/v) methanol |  | | 200 |  |
| 90% (v/v) methanol |  | | 250 |  |
| 10% (v/v) acetone, 80% (v/v) methanol | |  | 400 |  |
| 60% (v/v) acetone |  | | 200 |  |

**Table 2 -** Yields (%w/w) of the 5 GSE fractions obtained by gel permeation chromatography (GPC).

**Fractions yield (by weight)**

Fractions 1 5.5%

Fractions 2 2.9%

Fractions 3 9.4%

Fractions 4 21.6%

Fractions 5 48.8%

Total recovery 88.2%

# S.1.2. Fractions characterization by GPC Analysis

The fractions were analyzed by GPC according to the method described by Morazzoni P. *et al* [16].

Briefly, samples and standards were dissolved in THF/LiBr 12 mM (95:5) added of BHT as internal standard (0.3 mg/ml), vortexed for 30 seconds and centrifuged at 10,000 x g for 10 min, verifying the complete dissolution.

GPC measurements were performed using an HPLC system (Agilent 1260 Infinity II) equipped with a diode array detector set at 280 nm. A 5µm, 7.5 x 300 mm, 500 Å PLgel Individual Pore Size Column (Agilent PL1110-6525) connected to a pre-column filter 0.5 µm (Upchurch Scientific, WA, USA) was used at 15 °C for PACs separation, with an isocratic elution of THF/aqueous LiBr 12mM (95:5, v/v) at a flow rate of 1.0 mL/min. Injection volume was 10 µL, and the acquisition time 10 min., samples were injected at 1.0 mg/mL containing BHT 0.3 mg/ml as internal standard. To test the linearity of column response two different molecular weights, the 12-point calibration curve of certified polystyrene (MW range 152-56600 g/mol) was injected under the same conditions.

Two standards mix (EC, B1, tannic acid; ECG, tannic acid) were analyzed to obtain a specific calibration for proanthocyanidins. (−)-Epicatechin from 0.025 to 0.25 mg/ml was used for quantitation. The data were exported and processed using OriginPro software for graph creation.


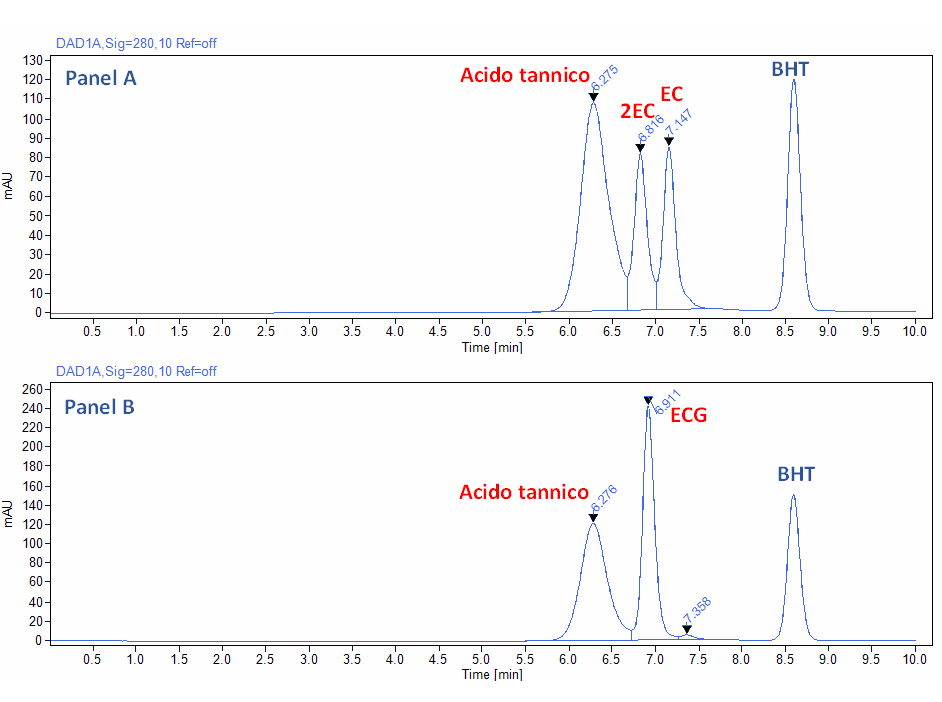


Tannic acid

Tannic acid

**Figure 1 -** GPC profiles of standards mix: epichatechin, procyanidin B1 and tannic acid (panel A); EGC and tannic acid (panel B).


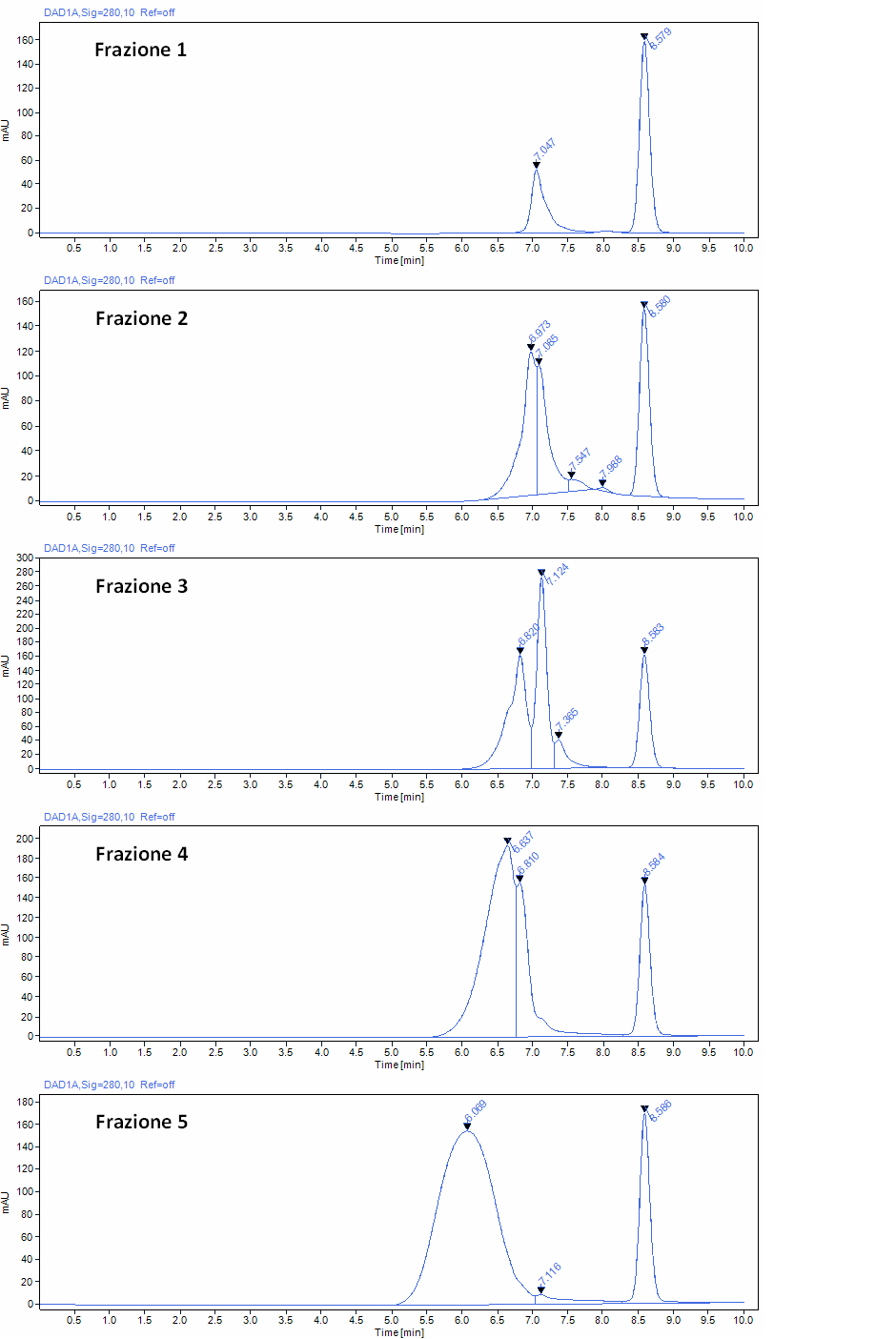


Fraction 1

Fraction 2

Fraction 3

Fraction 4

Fraction 5

**Figure 2 -** GPC profile of fractions 1, 2, 3, 4 and 5.


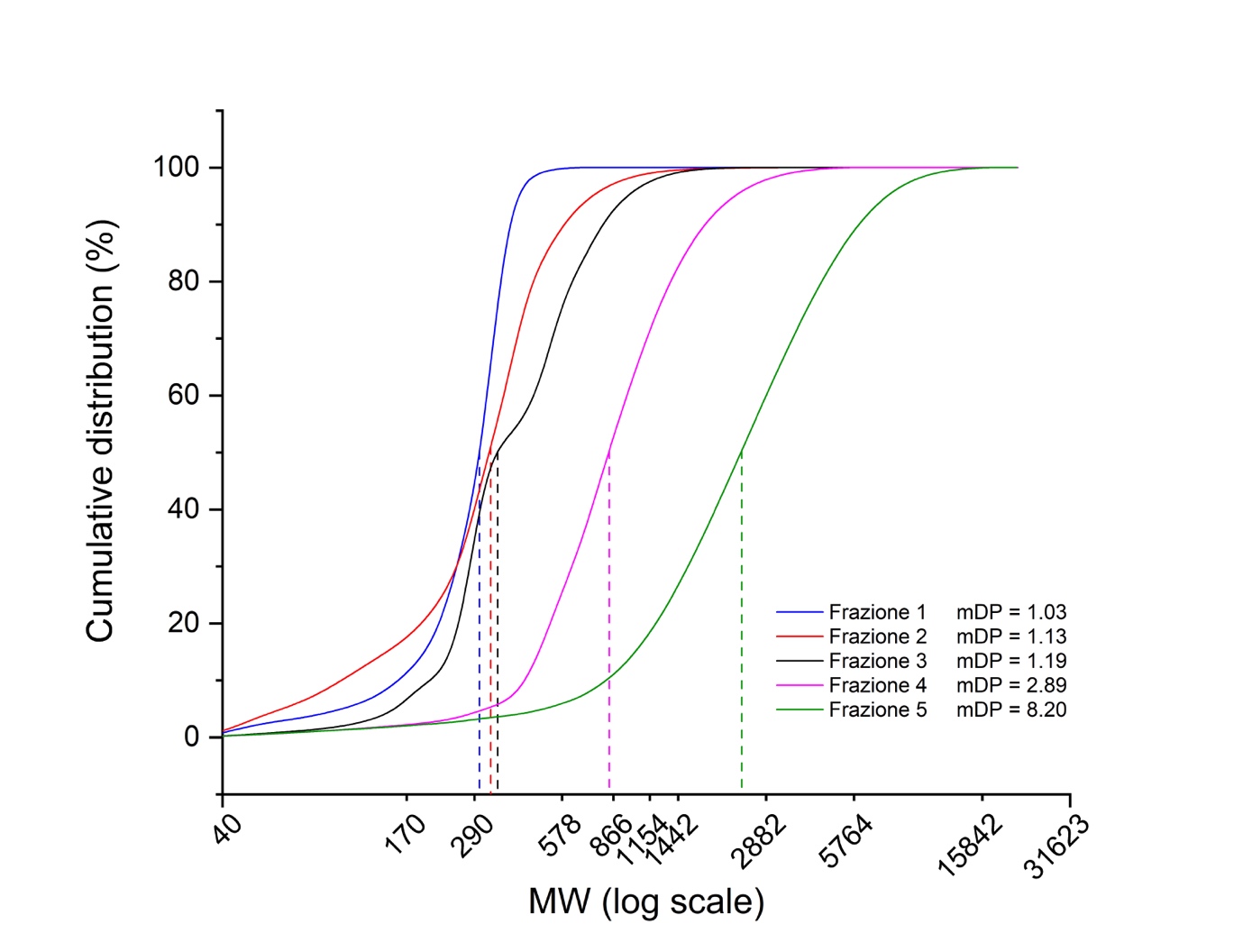


Fraction 1

Fraction 2

Fraction 3

Fraction 4

Fraction 5

**Figure 3 -** Cumulative distribution of proanthocyanidins in the fractions and mean degree of polymerization (mDP), expressed as the number of catechin units per average molecular weight [16].

# S.1.3. Fractions characterization by LC-MS analysis

LC-MS analysis of the five fractions was accomplished following the procedure previously described with minor modifications [16].

Sample preparation

Fractions 1-3 were dissolved at 1mg/ml in 0.2% acetic acid in water, while fractions 4-5 needed the addition of 10% acetone for complete dissolution. Before the MS analysis, the fractions were diluted with 0.08% formic acid water: methanol (60:40, v/v) solution to a final 20 µg/ml concentration.

LC-MS conditions

The flow injection analysis (FIA) mode was adopted on a 1200 series HPLC system through a Chip Cube nano-ESI interface coupled online with a 6520 Q-TOF mass spectrometer (Agilent Technologies, CA, USA) governed by Agilent MassHunter Workstation data acquisition software (B.05.00 version). Sample vials were placed at 10 °C in the autosampler compartment, and 2 µL were injected into an FIA-Chip (II) for the analysis. A solution 60:40 (v/v) of milli-Q water/ methanol with 0.08% formic acid was used as the mobile phase. The flow rate was 0.35 µL/min.

The Q-TOF operated in negative MS mode at 4 GHz high-resolution mode (R=20.000) with three reference mass correction ions to ensure mass accuracy. Data were acquired in profile full scan mode at a scan rate of 1 spectra/sec between 100 and 3200 *m/z*. The capillary voltage was set to 1600 V (negative polarity) with nitrogen as desolvating gas at 330 °C and 5 l/min; fragmentor, skimmer, and octupole were set at 250, 65, and 750 V, respectively.

In data-dependent MS/MS characterization, precursor ions were selected at an isolation width of 1.3 m/z and fragmented by collision-induced dissociation (CID) at fixed collision energies from 7 to 25 eV with an MS/MS scan rate of 1 spectrum s−1.

LC-MS data analysis

Data were processed with MassHunter Qualitative Analysis software release B07.00 (Agilent Technologies, Santa Clara, CA, USA). The resolved isotope deconvolution method of the Agilent MassHunter data system was used for the charge state deconvolution of the averaged mass spectra after background subtraction.

The Find by Molecular Feature Extraction (MFE) algorithm extracted compounds from the raw data. The algorithm uses the accuracy of the mass measurements to assign multiple species (ions) related to the same neutral molecule to a single compound, referred to as a feature. Negatively charged signals from deprotonation and formate adducts, with charged states up to 3, following the common molecules isotope model, were extracted. The extracted features list was then queried against the homemade PACs database of monoisotopic mass and chemical formulas (554 compounds) set to a 25-ppm tolerance.

Four base subunits (catechin, gallocatechin, catechin-3-O-gallate and gallocatechin-3-O-gallate) were utilized to create the theoretical PAC database of monoisotopic mass and chemical formulas. These four subunits represent the primary grape-derived PAC subunits. Theoretical oligomers from all possible combinations of the four units from the polymerization of 2 up to 15 were calculated, limiting the maximum number of EGC and galloylated bases to 3 and 5, respectively.

Features not identified by PAC database search were manually annotated on the MS/MS spectra base.

The sum of the individual ion abundances within the compound isotopic envelope was used as the parameter for compound abundance, expressed as a percentage of the total PACs ions. Previous study shows that this percentage corresponds to the molar fraction of the specific procyanidin [16].

**Fraction 1:**

In the fraction, unmodified procyanidins are absent. The specie with monoisotopic mass 456.1152 has been tentatively identified as epicatechin-3-*O* (3-*O*-methyl gallate), and the one with mass 472.1881 as epigallocatechin- (methyl gallate).

**Fraction 2:**

The main compounds are glucogallic acid (or galloyl glucose), about 50% (MW 332.079), (epi)catechin glycoside (MW 452.138), about 10% and 6% Gallic acid (MW 170.0224).

(epi)Catechin (MW 290.08) and dimeric procyanidin (2EC, MW 578,149) are present at 1-2% level. Compounds with MW 498.1420, 664.1575, 740.2031, and 784.2139 were not identified.

**Fraction 3:**

The fraction is characterized by 72% (epi)catechin (MW 290.08), 20% dimeric procyanidin (2EC, MW 578.15), 4% trimeric procyanidin (3EC, MW 866.23), <1% procyanidin 1EC1EGC (594), <1% procyanidin 2EC1EGC (604), and 3% gallic acid.

**Fraction 4:**

The fraction is characterized by 2% (epi)catechin (MW 290.08), 4% (epi)gallocatechin gallate (EGC, mW 458), 40% dimeric procyanidin (2EC, MW 578.15), 18% trimeric procyanidin (3EC, MW 866.23), 5% tetrameric procyanidin (4EC, MW 1154), and the remaining 26% consists of other species up to heptameric procyanidin (7EC) with many galloylated species ranging from 0.01-2% each.

**Fraction 5:**

The fraction is characterized by 8% (epi)catechin (MW 290.08), 5% (epi)catechin gallate (MW 442), 12% dimeric procyanidin (2EC, MW 578.15), 12% trimeric procyanidin (3EC, MW 866.23), 10% tetrameric procyanidin (4EC, MW 1154),3% hexameric procyanidin (6EC), 1.7% heptameric procyanidin (7EC) and the remaining 43% mainly consists of galloylated procyanidin from 3EC1G to 7EC1G ranging from 0.5-8% each.
