# Supplementary material for: Unraveling the parahormetic mechanism underlying the health-protecting effects of grapeseed procyanidins
Source: Redox Biol. 2023 Dec 7;69:102981. doi: 10.1016/j.redox.2023.102981 (PMC10770607; doi:10.1016/j.redox.2023.102981)
Supplement: Multimedia component 3 [file mmc3.docx]

**S.2. *In vitro* fermentation of grape seed extract fractions and UHPLC-MS/MS analysis of faecal catabolites**

**S.2.1. *In vitro* colonic fermentation procedure**

For the in vitro fermentation, 1 L of growth medium was prepared, aliquoted, and sterilized at 121 °C for 15 min in glass vessels (12 mL) before sample preparation. Fresh fecal samples were collected from three healthy donors with no previous intestinal diseases and who had not been treated with antibiotics for the previous 3 months. Donors also followed a strict controlled diet lacking polyphenol-containing foods for 2 days prior to fecal collection. Feces were immediately stored in anaerobic jars containing an anaerobic atmosphere generation bag to create an oxygen-free environment. An equal mass of feces from three donors was weighed and diluted with Dulbecco’s phosphate buffer saline (1%, w/v) and then homogenized to obtain a 10% (w/w) slurry to be used as the inoculum for fermentation. All the donors provided informed consent for the collection of fecal slurries. Blank samples containing the culture medium and the fecal slurry, without extracts, as well as abiotic control samples, containing the culture medium and the extracts, without fecal slurry, were prepared. An aqueous solution of every fraction (F1, F2, F3, F4, F5) was prepared to obtain a 75 μmol/L concentration in the final fermentation volume, based on the main hypothesized compound:

- epicatechin-3-*O*-methylgallate or epicatechin-3-*O*-vanillate (MW 456) for F1.

- glucogallic acid or galloyl-glucose (MW 332) for F2.

- epicatechin (MW 290) for F3.

- procyanidin tetramer (MW 1154) for F4.

- procyanidin heptamer (MW 2018) for F5.

Suspensions were left for 2 h at room temperature under constant magnetic stirring. In each fermentation batch, 45% of the growth medium, 45% of fecal slurry, and 10% of substrate suspension were added to reach a total fermentation volume of 4 mL. The fecal slurry and the aqueous substrate suspension were put into the vessel containing growth medium, were sealed, and flushed with N2 to create anaerobiosis. Vessels were incubated at 37 °C at 200 strokes min-1 in a Dubnoff bath (JULABO, Seelbach Germany). Samples were collected prior starting the fecal fermentation (0 h) and after 5 h and 24 h incubation. Microbial catabolism was stopped adding 10% v/v of acetonitrile, and samples were frozen (−80 °C) until extraction and analysis. All experiments were carried out in triplicate.

**S.2.2. Fecal metabolite extraction**

An aliquot of 300 µL of each fermented sample was extracted using 1200 μL of acidified methanol (0.1% v/v formic acid). The solution was vortexed for 2 min, sonicated for 10 min in an ultrasonic bath, vortexed for 2 min and re-sonicated for 5 min. Samples were centrifuged (Centrisart A-14C Refrigerated Micro-Centrifuge and Rotor YCSR-A1C, Sartorius Lab Instruments GmbH and Co. KG, Goettingen, Germany) at 14 460 × *g* for 10 min and the upper organic layer was transferred into a clean microfuge tube. After the first extraction, the residual pellet of the fermented samples was re-extracted following the same procedure, using 500 μL of the same solvent. Finally, supernatants were pooled. An aliquot of 1 mL of supernatant was dried under vacuum by centrifugal vacuum concentrator (SpeedVac Savant SPD121P, Thermo Fisher Scientific Inc., San Jos´e, CA, USA) and the dried pellet was re-suspended in 200 μL of acidified methanol (0.1% v/v formic acid), vortexed 2 min, sonicated 10 min and finally centrifuged 5 min at 14 460 × *g* at 4°C. The concentrated sample, as well as the remaining supernatant, were analyzed by uHPLC-ESI-MS/MS analysis.

**S.2.3. UHPLC-ESI-MS/MS Analysis**

The fecal extracts were analyzed using a UHPLC DIONEX Ultimate 3000 fitted with a TSQ Vantage triple quadrupole mass spectrometer (Thermo Fisher Scientific Inc., San Jose, CA, USA) equipped with a heated-electrospray ionization source (H-ESIII; Thermo Fisher Scientific Inc.). Separation was carried out by means of a Kinetex Evo C18 column (100x 2.1 mm; 2.6 µm particle size; Pheneomenex, CA, USA), installed with a precolumn cartridge (Phenomenex). Mobile phase, pumped at a flow-rate of 0.4 mL/min, consisted of a mixture of acidified water (0.01% v/v formic acid) (solvent A) and acidified acetonitrile (0.01% v/v formic acid) (solvent B). Following 0.5 min of 5% solvent B in A, the proportion of B was increased linearly to 40% over a period of 7 min. Solvent B was increased again to 80% in 1 min, maintained for 2 min and then the start conditions were re-established in 0.5 min and maintained for 3 min to re-equilibrate the column (total run: 14 min). The H-ESI-II interface was set to a capillary temperature of 275 °C and the source heater temperature was 250 °C. The sheath gas (N2) flow rate was set at 40 (arbitrary units), the auxiliary gas (N2) flow rate at 5 and the sweep gas flow was set at 15 (arbitrary units). The source voltage was 3 kV, the capillary voltage was -9 V and tube lens voltage was -53 V.

**Table 1 -** Retention time (RT) and selective reaction monitoring (SRM) conditions for identification and quantification of monomeric and oligomeric flavan-3-ols, gallic acid and their gut microbiota catabolites by UHPLC-ESI-MS/MS. nd and “-“ mean not detected. The nomenclature of gut microbiota catabolites was standardized according to Kay et al. (Am J Clin Nutr 112(4) (2020) 1051-1068). Nomenclature of fission catabolites are in accordance with Di Pede et al. [23].

|  |  |  |  | **Quantifier** | | **Qualifier** | |  |
| --- | --- | --- | --- | --- | --- | --- | --- | --- |
| **Compound (Abbreviation)** | **RT (min)** | **Parent ion**  **(M - H)^-^**  **(*m/z*)** | **S-lens** | **Product ion**  **(*m/z*)** | **CE (V)** | **Product ion**  **(*m/z*)** | **CE (V)** | **Standard used**  **for quantification** |
| **Parent compounds** | | | | | | | |  |
| **Monomeric flavan-3-ols** |  |  |  |  |  |  |  |  |
| (+)-Catechin (C) | 3.48 | 289 | 112 | 245 | 18 | 203 | 23 | C |
| (−)-Epicatechin (EC) | 4.25 | 289 | 112 | 245 | 18 | 203 | 23 | EC |
| (−)-Epigallocatechin (EGC) | - | 305 | 111 | 125 | 25 | 179 | 17 | n.d. |
| (−)-Epicatechin-3-*O*-gallate (ECG) | 4.79 | 441 | 86 | 289 | 20 | 169 | 21 | EGCG |
| (−)-Epigallocatechin-3-*O*-gallate (EGCG) | 4.36 | 457 | 86 | 169 | 21 | 125 | 44 | n.d. |
| **Monomeric modified flavan-3-ols** |  |  |  |  |  |  |  |  |
| (−)-Epicatechin-3-*O*-methylgallate or (−)-Epigallocatechin-3-*O*-vanillate | - | 455 | 98 | 288 | 20 | 440 | 25 | n.d. |
| (−)-Epigallocatechin-3-*O*-methylgallate | - | 471 | 86 | 304 | 20 | 456 | 25 | n.d. |
| **Oligomeric flavan-3-ols** |  |  |  |  |  |  |  |  |
| Dimer B2 (PC B2) | 4.15 | 577 | 131 | 289 | 27 | 407 | 25 | PC B2 |
| Dimer A2 (PC A2) | 4.86 | 575 | 131 | 289 | 25 | 449 | 25 | n.d. |
| Dimer type B (PC dimer B) | 3.23 | 577 | 131 | 289 | 27 | 407 | 25 | PC B2 |
| Trimer C1 (PC C1) | - | 865 | 140 | 865 | 25 | 289 | 25 | n.d. |
| Tetramer D (PC D) | 4.52 | 576* | 145 | 289 | 22 | 125 | 42 | PC D |
| Pentamer E | - | 720.6* | 204 | 289 | 34 | 125 | 45 | n.d. |
| **Fission catabolites** | | | | | | | |  |
| **Dimers** |  |  |  |  |  |  |  |  |
| 1 Fission Dimer B2 (PC B2_ 1 fission) | 4.73 | 579 | 131 | 291 | 25 | 289 | 25 | PC B2 |
| 2 Fission Dimer B2 (PC B2_ 2 fission) | 5.47 | 581 | 131 | 581 | 25 | 291 | 25 | PC B2 |
| 1 Fission Dimer A2 | - | 577 | 131 | 577 | 25 | 291 | 25 | n.d. |
| 2 Fission Dimer A2 | - | 579 | - | - | - | - | - | n.d. |
| **Trimers** |  |  |  |  |  |  |  |  |
| 1 Fission Trimer C1 | 4.65 | 867 | 140 | 867 | 25 | 291 | 25 | PC C1 |
| 2 Fission Trimer C1 | - | 869 | 140 | 869 | 25 | - | - | n.d. |
| 3 Fission Trimer C1 | - | 871 | - | - | - | - | - | n.d. |
| **Tetramers** |  |  |  |  |  |  |  |  |
| 1 Fission Tetramer D, form 1 | **-** | 578* | 145 | 291 | 25 | 578 | 25 | n.d. |
| 1 Fission Tetramer D, form 2 | **-** | 578* | 145 | 578 | 25 | 289 | 25 | n.d. |
| 2 Fission Tetramer D | - | 580* | 145 | 580 | 25 | 291 | 25 | n.d. |
| 3 Fission Tetramer D (PC D_3 fission) | 4.83 | 582* | 145 | 291 | 25 | 289 | 25 | PC D |
| 4 Fission Tetramer D | **-** | 584* | 145 | 584 | 25 | - | - | n.d. |
| **Diphenylpropan-2-ol derivatives (PPOLs)** |  |  |  |  |  |  |  |  |
| 1-(Hydroxyphenyl)-3-(2″,4″,6″-trihydroxyphenyl)-propan-2-ol | - | 275 | 98 | 231 | 16 | 191 | 30 | n.d. |
| 1-(3′,4′-Dihydroxyphenyl)-3-(2″,4″,6″-trihydroxyphenyl)-propan-2-ol (3’,4’-DiOH-PPOL) | 4.31 | 291 | 98 | 123 | 30 | 247 | 16 | 3′,4′-diOH-PVL |
| 1-(3′,5′-Dihydroxyphenyl)-3-(2″,4″,6″-trihydroxyphenyl)-propan-2-ol | - | 291 | 98 | 123 | 30 | 167 | 30 | n.d. |
| 1-(3′,4′,5′-Triihydroxyphenyl)-3-(2″,4″,6″-trihydroxyphenyl)-propan-2-ol | - | 307 | - | - | - | - | - | n.d. |
| 1-(3′,4′,5′-Trihydroxyphenyl)-3-(2″,4″,6″-trihydroxyphenyl) propan-2-yl gallate^§^ | - | 459 | - | - | - | - | - | n.d. |
| 1-(3′,4′-Dihydroxyphenyl)-3-(2″,4″,6″-trihydroxyphenyl)-propan-2-yl methylgallate | - | 457 | - | - | - | - | - | n.d. |
| 1-(3′,4′,5′-Triihydroxyphenyl)-3-(2″,4″,6″-trihydroxyphenyl)-propan-2-yl vanillate | - | 457 | - | - | - | - | - | n.d. |
| 1-(3′,4′,5′-Triihydroxyphenyl)-3-(2″,4″,6″-trihydroxyphenyl)-propan-2-yl methylgallate | - | 473 | - | - | - | - | - | n.d. |
| **Phenyl-γ-valerolactones (PVLs)** |  |  |  |  |  |  |  |  |
| 5-Phenyl-γ-valerolactone | - | 175 | - | - | - | - | - | n.d. |
| 5-(4′-Hydroxyphenyl)-γ-valerolactone | - | 191 | 67 | 147 | 16 | 106 | 31 | n.q. |
| 5-(3′-Hydroxyphenyl)-γ-valerolactone (3’-OH-PVL) | 5.02 | 191 | 67 | 147 | 16 | 106 | 31 | 3′-OH-PVL |
| 5-(3′,4′-Dihydroxyphenyl)-γ-valerolactone (3’,4’-DiOH-PVL or γ-V) | 4.25 | 207 | 81 | 163 | 18 | 122 | 21 | 3′,4′-diOH-PVL |
| 5-(3′,5′-Dihydroxyphenyl)-γ-valerolactone | - | 207 | 81 | 163 | 18 | 123 | 20 | n.d. |
| 5-(3′,4′,5′-Trihydroxyphenyl)-γ-valerolactone | - | 223 | 75 | 179 | 21 | 138 | 26 | n.d. |
| **Phenylvaleric acids (PVAs)** |  |  |  |  |  |  |  |  |
| 5-Phenylvaleric acid | - | 177 | - | - | - | - | - | n.d. |
| 5-(4′-Hydroxyphenyl)valeric acid | - | 193 | 71 | 147 | 30 | 175 | 30 | n.d. |
| 5-(3′-Hydroxyphenyl)valeric acid | - | 193 | 71 | 147 | 30 | 175 | 30 | n.d. |
| 4-Hydroxy-5-(phenyl)valeric acid | - | 193 | - | - | - | - | - | n.d. |
| 4-Hydroxy-5-(hydroxyphenyl)valeric acid (4-OH-3’/4’-OH-PVA) | 4.38 | 209 | 63 | 147 | 20 | 101 | 20 | 3′,4′-diOH-PVL |
| 5-(3′,4′-Dihydroxyphenyl)valeric acid (3’,4’-DiOH-PVA) | 5.02 | 209 | 63 | 191 | 15 | 165 | 12 | 3′,4′-diOH-PVL |
| 5-(3′,5′-Dihydroxyphenyl)valeric acid | - | 209 | 63 | 191 | 15 | 147 | 20 | n.d. |
| 4-Hydroxy-5-(3′,4′-Dihydroxyphenyl)valeric | - | 225 | - | - | - | - | - | n.d. |
| 4-Hydroxy-5-(3′,5′-Dihydroxyphenyl)valeric acid | - | 225 | - | - | - | - | - | n.d. |
| 5-(3′,4′,5′-Trihydroxyphenyl)valeric acid | - | 225 | - | - | - | - | - | n.d. |
| 4-Hydroxy-5-(3′,4′,5′-Trihydroxyphenyl)valeric acid | - | 241 | - | - | - | - | - | n.d. |
| **Phenylpropanoic acids (PPAs)** |  |  |  |  |  |  |  |  |
| 3-Phenylpropanoic acid (PPA) | 5.06 | 149 | 60 | 149 | 10 | 105 | 13 | n.d. |
| 3-(4′-Hydroxyphenyl)propanoic acid (4’-HPPA) | 4.34 | 165 | 64 | 121 | 13 | 93 | 14 | n.d. |
| 3-(3′-Hydroxyphenyl)propanoic acid (3’-HPPA) | 4.61 | 165 | 64 | 121 | 13 | 119 | 18 | 3′-OH-PPA |
| 2-Hydroxy-3-(phenyl)propanoic acid | - | 165 | - | - | - | - | - | n.d. |
| 3-(3′,4′-Dihydroxyphenyl)propanoic acid (3’,4’-DiHPPA) | 3.25 | 181 | 64 | 137 | 14 | 109 | 18 | n.d. |
| 3-(3′,5′-Dihydroxyphenyl)propanoic acid | - | 181 | 64 | 137 | 14 | - | - | n.d. |
| 2-Hydroxy-3-(hydroxyphenyl)propanoic acid | - | 181 | - | - | - | - | - | n.d. |
| 3-(4′-Hydroxy-3′-methoxyphenyl)propanoic acid | - | 195 | - | - | - | - | - | n.d. |
| 3-(3′,4′,5′-Trihydroxyphenyl)propanoic acid | - | 197 | - | - | - | - | - | n.d. |
| **Phenylacetic acids (PAAs)** |  |  |  |  |  |  |  |  |
| Phenylacetic acid | 5.04 | 135 | 40 | 91 | 10 | 135 | 10 | n.d. |
| 4′-Hydroxyphenylacetic acid | 3.14 | 151 | 75 | 107 | 13 | - | - | n.d. |
| 3′-Hydroxyphenylacetic acid | 3.77 | 151 | 75 | 107 | 13 | - | - | n.d. |
| 3′,4′-Dihydroxyphenylacetic acid | 1.84 | 167 | 35 | 123 | 13 | 122 | 28 | n.d. |
| 3′,4′,5′-Trihydroxyphenylacetic acid | **-** | 183 | - | - | - | - | - | n.d. |
| **Benzoic acids (BAs)** |  |  |  |  |  |  |  |  |
| Benzoic acid | 4.96 | 121 | 68 | 77 | 13 | 121 | 10 | n.d. |
| 4-Hydroxybenzoic acid | 2.62 | 137 | 72 | 93 | 16 | - | - | n.d. |
| 3-Hydroxybenzoic acid | 3.63 | 137 | 72 | 93 | 16 | - | - | n.d. |
| 3,4-Dihydroxybenzoic acid | 1.54 | 153 | 74 | 109 | 16 | 108 | 26 | n.d. |
| 3,5-Dihydroxybenzoic acid | - | 153 | - | - | - | - | - | n.d. |
| 3,4,5-Trihydroxybenzoic acid (3,4,5-TriOH-BA or gallic acid) | 0.92 | 169 | 78 | 125 | 17 | 79 | 25 | 3,4,5-TriOH-BA |
| **Benzaldehydes (BALs)** |  |  |  |  |  |  |  |  |
| 4-Hydroxybenzaldehyde | 3.68 | 121 | 68 | 92 | 26 | 120 | 20 | n.d. |
| 3,4-Dihydroxybenzaldehyde | 2.30 | 137 | 72 | 136 | 22 | 108 | 26 | n.d. |
| 2,4,6-Trihydroxybenzaldeyde | - | 153 | - | - | - | - | - | n.d. |
| **Benzene derivatives (BZs)** |  |  |  |  |  |  |  |  |
| Hydroxybenzyl alcohol | - | 123 | - | - | - | - | - | n.d. |
| Benzene-1,2-diol (1,2-DiOH-BZ or catechol) | 1.52 | 109 | 62 | 108 | 10 | - | - | 1,2-DiOH-BZ |
| Benzene-1,2,3-triol (1,2,3-TriOH-BZ or pyrogallol) | 1.03 | 125 | 62 | 125 | 10 | 79 | 22 | 1,2,3-TriOH-BZ |

**Table 2** **-** Compounds identified and quantified in the samples following fecal fermentation.

| **Parent compounds** | **Microbial catabolites** |
| --- | --- |
| ***Monomeric flavan-3-ols*** | ***Fission catabolites*** |
| (+)-Catechin (C) | 1 Fission Dimer B2 |
| (−)-Epicatechin (EC) | 2 Fission Dimer B2 |
| (−)-Epicatechin-3-*O*-gallate (ECG) | 1 Fission Trimer C1 |
| **Oligomeric flavan-3-ols** | 3 fission tetramer D |
| procyanidin dimer B2 | ***Diphenylpropan-2-ol*** |
| procyanidin dimer type B | 1-(3′,4′-dihydroxyphenyl)-3-(2″,4″,6″-trihydroxyphenyl)-propan-2-ol |
| Tetramer D | ***Phenyl-γ-valerolactones*** |
| ***Phenolic acid*** | 5-(3′-hydroxyphenyl)-γ-valerolactone |
| Gallic acid | 5-(3′,4′-dihydroxyphenyl)-γ-valerolactone |
|  | ***Phenylvaleric acids*** |
|  | 4-hydroxy-5-(hydroxyphenyl)valeric acid |
|  | 5-(3′,4′-dihydroxyphenyl)valeric acid |
|  | ***Phenylpropanoic acid*** |
|  | 3-(3′-hydroxyphenyl)propanoic acid |
|  | ***Benzoic acid*** |
|  | 3,4,5-trihydroxybenzoic acid |
|  | ***Benzene derivatives*** |
|  | benzene-1,2,3-triol |
|  | benzene-1,2-diol |

**Table 3 -** Quantification of parent compounds and microbial catabolites of F1 in different fermentation times (T0, T5, T24). Data are reported as mean ± standard deviation (µmol/L). Different low case letters indicate significant differences among different incubation times (p<0.05).

| **Compound** | **T0 (µmol/L)** | **T5 (µmol/L)** | **T24 (µmol/L)** |
| --- | --- | --- | --- |
| 3’,4’-DiOH-PVL | 0.0 ± 0.0 ^b^ | 0.08 ± 0.0 ^a^ | 0.0 ± 0.0 ^b^ |
| 3,4,5-TriOH-BA | 0.15 ± 0.01 ^a^ | 0.17 ± 0.02 ^a^ | 0.0 ± 0.0 ^b^ |

**Table 4 -** Quantification of parent compounds and microbial catabolites of F2 in different fermentation times (T0, T5, T24). Data are reported as mean ± standard deviation (µmol/L). Different low case letters indicate significant differences among different incubation times (p<0.05).

| **Compound** | **T0 (µmol/L)** | **T5 (µmol/L)** | **T24 (µmol/L)** |
| --- | --- | --- | --- |
| PC B2_ 1 fission | 0.0 ± 0.0 ^b^ | 0.0 ± 0.0 ^b^ | 0.07 ± 0.01 ^a^ |
| 3’,4’-DiOH-PPOL | 0.0 ± 0.0 ^b^ | 0.39 ± 0.09 ^a^ | 0.0 ± 0.0 ^b^ |
| 3’-OH-PVL | 0.0 ± 0.0 ^b^ | 0.0 ± 0.0 ^b^ | 0.90 ± 0.14 ^a^ |
| 3’,4’-DiOH-PVL | 0.0 ± 0.0 ^c^ | 5.12 ± 0.04 ^b^ | 9.38 ± 0.29 ^a^ |
| 4-OH-3’/4’-OH-PVA | 0.0 ± 0.0 ^c^ | 0.04 ± 0.0 ^b^ | 0.13 ± 0.01 ^a^ |
| 3’,4’-DiOH-PVA | 0.0 ± 0.0 ^b^ | 0.0 ± 0.0 ^b^ | 0.15 ± 0.03 ^a^ |
| 3’-HPPA | 0.0 ± 0.0 ^b^ | 0.0 ± 0.0 ^b^ | 1.80 ± 0.58 ^a^ |
| 3,4,5-TriOH-BA | 14.68 ± 1.04 ^a^ | 16.0 ± 4.36 ^a^ | 0.0 ± 0.0 ^b^ |
| 1,2,3-TriOH-BZ | 0.0 ± 0.0 ^b^ | 6.11 ± 0.38 ^a^ | 0.0 ± 0.0 ^b^ |
| 1,2-DiOH-BZ | 0.0 ± 0.0 ^b^ | 0.78 ± 0.06 ^a^ | 0.0 ± 0.0 ^b^ |

**Table 5 -** Quantification of parent compounds and microbial catabolites of F3 in different fermentation times (T0, T5, T24). Data are reported as mean ± standard deviation (µmol/L). Different low case letters indicate significant differences among different incubation times (p<0.05).

| **Compound** | **T0 (µmol/L)** | **T5 (µmol/L)** | **T24 (µmol/L)** |
| --- | --- | --- | --- |
| C | 10.55 ± 0.59 ^a^ | 0.0 ± 0.0 ^b^ | 0.0 ± 0.0 ^b^ |
| EC | 4.73 ± 0.24 ^a^ | 0.74 ± 0.06 ^b^ | 0.0 ± 0.0 ^c^ |
| PC B2 | 0.41 ± 0.13 ^a^ | 0.0 ± 0.0 ^b^ | 0.0 ± 0.0 ^b^ |
| PC dimer B | 0.09 ± 0.0 ^a^ | 0.0 ± 0.0 ^b^ | 0.0 ± 0.0 ^b^ |
| PC B2_ 1 fission | 0.0 ± 0.0 ^c^ | 0.52 ± 0.07 ^a^ | 0.12 ± 0.01 ^b^ |
| PC B2_ 2 fission | 0.0 ± 0.0 ^b^ | 0.0 ± 0.0 ^b^ | 0.31 ± 0.03 ^a^ |
| PC D_3 fission | 0.0 ± 0.0 ^b^ | 0.08 ± 0.01 ^a^ | 0.0 ± 0.0 ^b^ |
| 3’,4’-DiOH-PPOL | 0.0 ± 0.0 ^b^ | 0.24 ± 0.06 ^a^ | 0.0 ± 0.0 ^b^ |
| 3’-OH-PVL | 0.0 ± 0.0 ^c^ | 2.30 ± 0.24 ^a^ | 2.78 ± 0.06 ^b^ |
| 3’,4’-DiOH-PVL | 0.0 ± 0.0 ^c^ | 55.93 ± 3.76 ^a^ | 45.97 ± 0.11 ^b^ |
| 4-OH-3’/4’-OH-PVA | 0.0 ± 0.0 ^c^ | 0.83 ± 0.07 ^a^ | 0.64 ± 0.05 ^b^ |
| 3’,4’-DiOH-PVA | 0.0 ± 0.0 ^b^ | 0.17 ± 0.04 ^b^ | 1.81 ± 0.19 ^a^ |
| 3’-HPPA | 0.0 ± 0.0 ^c^ | 0.81 ± 0.15 ^b^ | 2.45 ± 0.41 ^a^ |
| 3,4,5-TriOH-BA | 4.43 ± 0.18 ^a^ | 0.50 ± 0.05 ^b^ | 0.0 ± 0.0 ^c^ |
| 1,2,3-TriOH-BZ | 0.0 ± 0.0 ^b^ | 0.83 ± 0.09 ^a^ | 0.0 ± 0.0 ^b^ |

**Table 6 -** Quantification of parent compounds and microbial catabolites of F4 in different fermentation times (T0, T5, T24). Data are reported as mean ± standard deviation (µmol/L). Different low case letters indicate significant differences among different incubation times (p<0.05).

| **Compound** | **t0 (µmol/L)** | **t5 (µmol/L)** | **t24 (µmol/L)** |
| --- | --- | --- | --- |
| C | 1.04 ± 0.13 ^a^ | 0.0 ± 0.0 ^b^ | 0.0 ± 0.0 ^b^ |
| EC | 1.29 ± 0.19 ^a^ | 0.65 ± 0.09 ^b^ | 0.63 ± 0.04 ^b^ |
| ECG | 2.60 ± 0.19 ^a^ | 0.86 ± 0.05 ^b^ | 0.07 ± 0.01 ^c^ |
| PC B2 | 1.97 ± 0.08 ^a^ | 0.03 ± 0.0 ^b^ | 0.14 ± 0.03 ^b^ |
| PC dimer B | 1.06 ± 0.09 ^a^ | 0.0 ± 0.0 ^b^ | 0.0 ± 0.0 ^b^ |
| PC D | 0.47 ± 0.06 ^a^ | 0.03 ± 0.02 ^c^ | 0.15 ± 0.03 ^b^ |
| PC B2_ 1 fission | 0.0 ± 0.0 ^c^ | 4.88 ± 0.32 ^a^ | 1.38 ± 0.08 ^b^ |
| PC B2_ 2 fission | 0.0 ± 0.0 ^b^ | 0.0 ± 0.0 ^b^ | 4.11 ± 0.66 ^a^ |
| PC C1_1 fission | 0.0 ± 0.0 ^b^ | 4.61 ± 0.37 ^a^ | 0.0 ± 0.0 ^b^ |
| PC D_3 fission | 0.0 ± 0.0 ^b^ | 0.78 ± 0.03 ^a^ | 0.80 ± 0.02 ^a^ |
| 3’,4’-DiOH-PPOL | 0.0 ± 0.0 ^c^ | 0.64 ± 0.02 ^a^ | 0.45 ± 0.03 ^b^ |
| 3’-OH-PVL | 0.0 ± 0.0 ^c^ | 1.90 ± 0.44 ^b^ | 3.05 ± 0.23 ^a^ |
| 3’,4’-DiOH-PVL | 0.0 ± 0.0 ^b^ | 60.41 ± 12.40 ^a^ | 55.74 ± 2.60 ^a^ |
| 4-OH-3’/4’-OH-PVA | 0.0 ± 0.0 ^c^ | 0.24 ± 0.07 ^b^ | 0.49 ± 0.08 ^a^ |
| 3’,4’-DiOH-PVA | 0.0 ± 0.0 ^b^ | 0.04 ± 0.0 ^b^ | 1.66 ± 0.20 ^a^ |
| 3’-HPPA | 0.0 ± 0.0 ^b^ | 0.52 ± 0.13 ^b^ | 2.98 ± 0.69 ^a^ |
| 3,4,5-TriOH-BA | 4.07 ± 0.20 ^b^ | 5.45 ± 0.69 ^a^ | 0.58 ± 0.04 ^c^ |
| 1,2,3-TriOH-BZ | 0.0 ± 0.0 ^b^ | 2.16 ± 0.12 ^a^ | 0.0 ± 0.0 ^b^ |

**Table 7 -** Quantification of parent compounds and microbial catabolites of F5 in different fermentation times (T0, T5, T24). Data are reported as mean ± standard deviation (µmol/L). Different low case letters indicate significant differences among different incubation times (p<0.05).

| **Compound** | **t0 (µmol/L)** | **t5 (µmol/L)** | **t24 (µmol/L)** |
| --- | --- | --- | --- |
| C | 0.23 ± 0.07 ^a^ | 0.0 ± 0.0 ^b^ | 0.0 ± 0.0 ^b^ |
| EC | 0.59 ± 0.01 ^a^ | 0.46 ± 0.02 ^b^ | 0.0 ± 0.0 ^c^ |
| ECG | 0.23 ± 0.02 ^a^ | 0.09 ± 0.01 ^b^ | 0.0 ± 0.0 ^c^ |
| PC B2 | 0.18 ± 0.04 ^a^ | 0.0 ± 0.0 ^b^ | 0.0 ± 0.0 ^b^ |
| PC B2_ 1 fission | 0.0 ± 0.0 ^c^ | 0.62 ± 0.05 ^a^ | 0.23 ± 0.0 ^b^ |
| PC B2_ 2 fission | 0.0 ± 0.0 ^b^ | 0.0 ± 0.0 ^b^ | 1.03 ± 0.01 ^a^ |
| PC D_3 fission | 0.0 ± 0.0 ^b^ | 0.08 ± 0.01 ^a^ | 0.11 ± 0.03 ^a^ |
| 3’,4’-DiOH-PPOL | 0.0 ± 0.0 ^b^ | 0.44 ± 0.08 ^a^ | 0.0 ± 0.0 ^b^ |
| 3’-OH-PVL | 0.0 ± 0.0 ^b^ | 0.0 ± 0.0 ^b^ | 1.46 ± 0.44 ^a^ |
| 3’,4’-DiOH-PVL | 0.0 ± 0.0 ^b^ | 10.29 ± 0.96 ^a,b^ | 25.59 ± 7.66 ^a^ |
| 4-OH-3’/4’-OH-PVA | 0.0 ± 0.0 ^c^ | 0.05 ± 0.0 ^b^ | 0.18 ± 0.02 ^a^ |
| 3’,4’-DiOH-PVA | 0.0 ± 0.0 ^b^ | 0.0 ± 0.0 ^b^ | 0.54 ± 0.11 ^a^ |
| 3’-HPPA | 0.0 ± 0.0 ^b^ | 0.90 ± 0.22 ^b^ | 4.14 ± 1.02 ^a^ |
| 3,4,5-TriOH-BA | 3.55 ± 0.05 ^a^ | 1.52 ± 0.06 ^b^ | 0.47 ± 0.02 ^c^ |
| 1,2,3-TriOH-BZ | 0.0 ± 0.0 ^b^ | 0.91 ± 0.14 ^a^ | 0.0 ± 0.0 ^b^ |
