# Supplementary material for: Unraveling the parahormetic mechanism underlying the health-protecting effects of grapeseed procyanidins
Source: Redox Biol. 2023 Dec 7;69:102981. doi: 10.1016/j.redox.2023.102981 (PMC10770607; doi:10.1016/j.redox.2023.102981)
Supplement: Multimedia component 4 [file mmc4.docx]

**S.3. Intervention study: Experimental design**

**

**Figure 1** **-** Schematic representation of the pilot study.

**S.3.1. Baseline characteristic of the study population**

One participant dropped out from the study for personal reasons, not related to the study. Thus, 11 healthy subjects (M/F, 4/7; age 29±4.8 y; BMI 21.2±2.1 kg/m^2^) successfully completed the trial. All the subjects were healthy and no abnormalities in the main biochemical parameters were observed.

**S.3.2. Compliance**

Subjects were highly motivated to participate to the trial and confirmed the consumption of the tablets and the adherence to the dietary instructions. The compliance was verified with a direct interview, with the collection of the food diaries, and by returning the blisters of the supplements.
