# Supplementary material for: Unraveling the parahormetic mechanism underlying the health-protecting effects of grapeseed procyanidins
Source: Redox Biol. 2023 Dec 7;69:102981. doi: 10.1016/j.redox.2023.102981 (PMC10770607; doi:10.1016/j.redox.2023.102981)
Supplement: Multimedia component 5 [file mmc5.docx]

**S.4. Urinary and plasma metabolites identification by LC-MS**

**S.4.1. LC.MS/MS conditions**

The chromatographic separation was performed by a Exion LC 100 system (AB Sciex, Milan, Italy) equipped with a quaternary pump, working at a constant flow rate of 0.4 mL/min of mobile phase A (H_2_O-0.2% HCOOH, %v/v) and mobile phase B (CH_3_CN-0.2% HCOOH, %v/v) with the following multi-step gradient program: from 5 % B to 55 % B in 20 min, from 55 % B to 75 % B in 1 min, isocratic of 75 % B for 4 min, then isocratic of 5 % B for 6 min. The LC was connected to an API 4000 triple quadrupole mass spectrometer (AB Sciex, Milan, Italy), equipped with a TurboV electrospray interface (AB Sciex, Milan, Italy), operating in negative ion mode by applying -4.5 kV ionization potential, 25 units of curtain gas, 40 units of gas 1, 10 units of gas 2 heated at 400 °C. The mass spectrometer conditions for the MRM analysis were optimized by direct infusion of standard solution (10 µM) of the valerolactones mixture into the source. Two transitions for each analyte were selected, one for the quantitative analysis and the second one as qualitative confirmation of the compound (Table 1).

**Table 1 -** Optimized MRM transitions for valerolactone metabolites

| **Compound name** | **Parent ion**  **(*m/z*)** | **Product ions**  **(*m/z*)** | |
| --- | --- | --- | --- |
| 5-(3´-hydroxyphenyl)-γ-valerolactone-4´-glucuronide (1) | 383 | 207 | 113 |
| 5-(3´-hydroxyphenyl)-γ-valerolactone-4´-sulfate (2) | 287 | 207 | 163 |
| 5-(3´,4´-dihydroxyphenyl)-γ-valerolactone (3) | 207 | 163 | 122 |
| 5-(3’,4’-dihydroxyphenyl)-γ-valerolactone D4 (IS) | 211 | 167 | 122 |
