# Supplementary material for: Unraveling the parahormetic mechanism underlying the health-protecting effects of grapeseed procyanidins
Source: Redox Biol. 2023 Dec 7;69:102981. doi: 10.1016/j.redox.2023.102981 (PMC10770607; doi:10.1016/j.redox.2023.102981)
Supplement: Multimedia component 6 [file mmc6.docx]

**S.5. Cell studies**

**S.5.1 Anti-inflammatory and antioxidant activity in gene-reporter cells**

*S.5.1.1 Anti-oxidant activity in HEK293 cells*

Experiments were performed using Nrf2/ARE Responsive Luciferase Reporter HEK293 stable cell line (Signosis, Santa Clara, CA, USA) in Dulbecco modified Eagle medium (DMEM; Lonza, Verviers, Belgium) supplemented with 10% fetal bovine serum (FBS; Gibco, Gaithersburg, MD, USA), 1 % Penicillin/Streptomycin (Lonza) and 50 mg/mL of G418 sulfate solution (Promega Corporation, Madison, WI, USA). HEK293 cells were treated with different concentrations of VL and bardoxolone (BDX) as Nrf2 std activator for 18 h after seeding in white 96-well plate (BRANDplates®, cell grade) at 1x10^4^ cells/well. Subsequently, to avoid any interference on the reading of luciferase activity, medium was removed and 100 µL/well of warm PBS was added. ONE-Glo™ Luciferase Assay Substrate (purchased from Promega Corporation, Madison, WI, USA) (100 µL/well) was directly added to the wells, followed by a luciferase measurement performed using a luminometer (Wallac Victor2 1420, Perkin-Elmer™ Life Science, Monza, Italy). Experiments were performed with biological and technical replicates; The cell viability was assessed with the MTT assay on HEK293 cells.

*
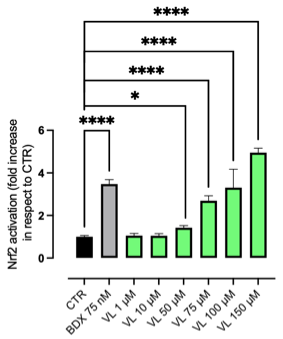
*

**Figure 1** **-** Dose dependent Nrf2 activation by 5-(3’,4’-dihydroxyphenyl)-γ-valerolactone (VL). Data are reported as mean ± SD. The statistical significance difference of each relative abundance has been analyzed by ONE-WAY ANOVA analysis followed by Tukey’s multiple comparisons test with 95% confidence interval. *p<0.05; ****p<0.0001. CTRL: control cells; BDX: bardoxolone.

*S.5.1.2 Anti-inflammatory activity R3/1*

The ability of VL to act as anti-inflammatory agent was tested in R3/1 cell lines by evaluating the NF-κB nuclear translocation. Cells were seeded in a white 96-well plate (BRANDplates^®^ cell grade) at a density of 4x10^3^ cells/well. Cells were pre-treated with different concentration of VL for 18 h in complete medium (DMEM 10 % FBS, 1 % L-glutamine, 1 % Penicillin/Streptomycin) and then stimulated with 10 ng/mL TNFα for 6 hrs. To avoid components interference on the reading of the luciferase assay, cells were washed once with 100 µL of warm PBS and 100 µL of DMEM were added. Subsequently, 100 µL ONE-Glo™ Luciferase Assay Substrate (purchased from Promega Corporation, Madison, WI, USA) was directly added to the wells, followed by a luciferase measurement performed using a luminometer (Wallac Victor2 1420, Perkin-Elmer™ Life Science, Monza, Italy). Experiments were performed with biological and technical replicates.

**Figure 2** **-** Effect of 5-(3',4'-dihydroxyphenyl)-γ-valerolactone (VL) in inhibiting NF-κB activation induced by the TNFa stimulus. Data are reported as mean ± SD. The statistical significance difference of each relative abundance has been analyzed by ONE-WAY ANOVA analysis followed by Tukey’s multiple comparisons test with 95% confidence interval.

**S.5.2. Anti-inflammatory activity in human intestinal Caco-2 cells**

*S.5.2.1. MTT (3-(4,5-Dimethylthiazol-2-yl)-2,5-Diphenyltetrazolium Bromide) Assay*

MTT assay was conducted following the method of Lammi C. et al. [30]. In a 96-well, Caco-2 cells (2.5 × 10^3^) were seeded and treated with VL (25 to 200 µM), or vehicle in complete DMEM for 48 h at 37 °C under 5% CO_2_ atmosphere. After incubation, the treatment was eliminated and 100 µL/well of MTT (0.5 mg/mL) filtered solution added. After 2 h of incubation at 37 °C under 5% CO_2_ atmosphere, the MTT solution was aspirated and 100 µL/well of the lysis buffer (8 mM HCl + 0.5% NP-40 in DMSO) added. After 10 min of slow shaking, the Synergy H1 microplate reader (Biotek, Bad Friedrichshall, Germany) was applied to read the absorbance at 570/630 nm.

**
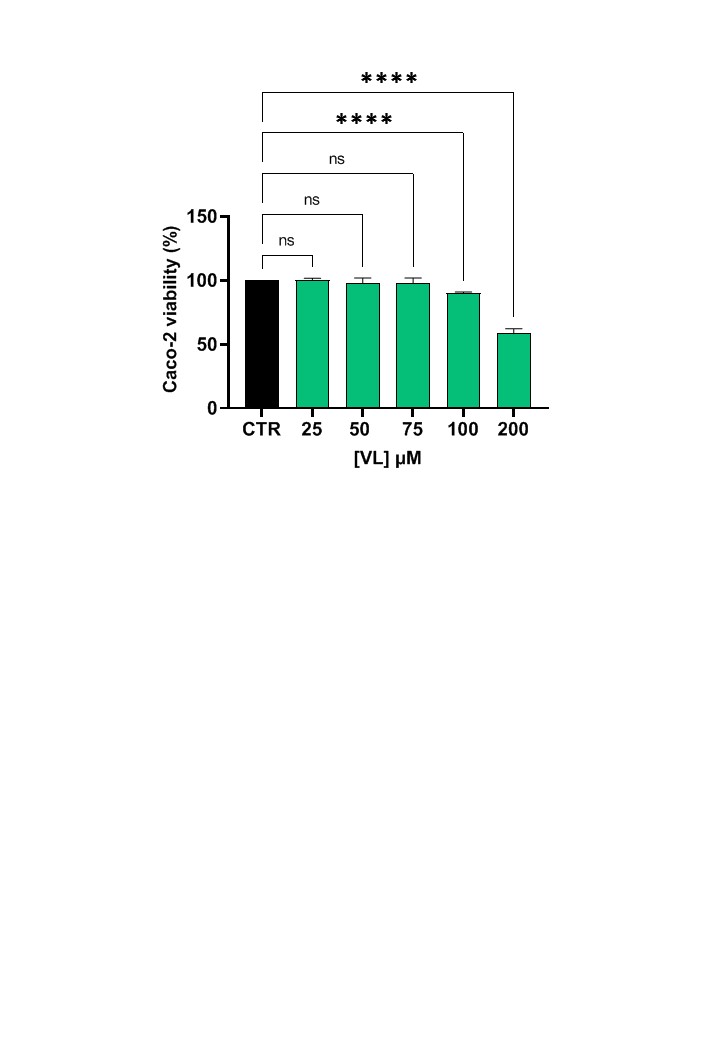
**

**Figure 3 -** Effect of VL on Caco-2 cells viability. Bar graphs indicating the results of cell viability assay of Caco-2 cells after VL (25-75 μM) treatment for 48 h. The data points represent the averages ± SD of three experiments in triplicate, statistical analysis was performed by one-way ANOVA. C: control sample, ns: not significant.

*S.5.2.2 Western Blot analysis*

Caco-2 cells (1.5 x 10^4^ well) were seeded on 24-well plates and incubated at 37 °C under 5% CO_2_ atmosphere. The next day, cells were treated with 350 µL/well of VL at a final concentration of 50 µM in complete growth medium for 24 h. The day after, cells were incubated with 15 ng/mL of TNFα or vehicle for 24 h. Cellular proteins were obtained from Caco-2 cells at at the maximum confluence of 50%, using 30 µL ice‐cold lysis buffer (RIPA buffer + inhibitor cocktail + 1:100 PMSF + 1:100 Na‐orthovanadate + 1:1000 β-mercaptoethanol). Bradford’s method was applied to quantify the total protein content and 40 μg of total proteins loaded on a pre‐cast 7.5% SDS‐PAGE gel at 130 V for 50 min. Subsequently, the gel was pre‐equilibrated in H_2_O for 5 min at room temperature (RT) and transferred to a nitrocellulose membrane (Mini nitrocellulose Transfer Packs,) using a Trans‐Blot Turbo at 1.3 A, 25 V for 7 min. Thereafter, the membranes were blocked with 5% milk or BSA and incubated overnight at 4°C with primary antibodies anti-NF-κB, anti- phospho(ser276)-NF-κB and anti‐tubulin. The next day, the membranes were washed and incubated with secondary antibodies conjugated with HRP for 1h at RT and a chemiluminescent reagent was used to visualize target proteins. Their signal was quantified using the Image Lab Software (Biorad, Hercules, CA). The internal control tubulin was used to normalize loading variations.

*S.5.2.3 Relating in vitro to ex vivo VL amounts*

The antioxidant and anti-inflammatory activities as found in Caco-2 cells was achieved by using a concentration of 50 µM. To evaluate whether the absorbed amount of VL (37 µmoles excreted in 24 hours) can reach the active concentration as found in in vitro conditions (50 µM), we made the following assumptions and calculations. The total area of colonocytes in the colon can be estimated as 14,650 cm^2^, a value calculated by considering a colon length of 1.5 mt, a diameter of 4.8 cm and surface amplification due to microvilli of 6.5 fold (Herbert F Helander & Lars Fändriks (2014) Surface area of the digestive tract – revisited, Scandinavian Journal of Gastroenterology, 49:6, 681-689, DOI: 10.3109/00365521.2014.898326). The amount of VL absorbed within 24 hours can be calculated by summing the amounts of excreted metabolites of the parent compound which reach a mean value of 37 µmoles. Hence a surface of one cm^2^ of colonocytes is exposed to 2.52 nmoles of VL in 24 hours after a dose of 300 mg of GSE. In vitro experiments were carried out using 24 well plates with a surface of 1,9 cm^2^ each well. Each well was loaded with 0.35 ml of medium. Taking into account the concentration of 50 nmoles/ml incubated and that the experiments on colonocytes lasted 48 hours, the amount of active VL in colonocytes was 4,6 nmoles per cm^2^ per 24 hours. Extrapolating the amount of excreted VL, we can assume that within 24 hours the dose of 300 mg of GSE, VL reaches the colonocytes at a concentration of 2.52 nmoles cm^2^, a value which is in the same order of magnitude in respect to that able to exert a robust and significant cellular protective activity as found in in vitro experiments: 4.6 nmoles per cm^2^ per 24 hours.
