# Supplementary material for: Unraveling the parahormetic mechanism underlying the health-protecting effects of grapeseed procyanidins
Source: Redox Biol. 2023 Dec 7;69:102981. doi: 10.1016/j.redox.2023.102981 (PMC10770607; doi:10.1016/j.redox.2023.102981)
Supplement: Multimedia component 7 [file mmc7.docx]

**S.6. Proteomic studies**

**S.6.1 Experimental design for proteomic studies**

**
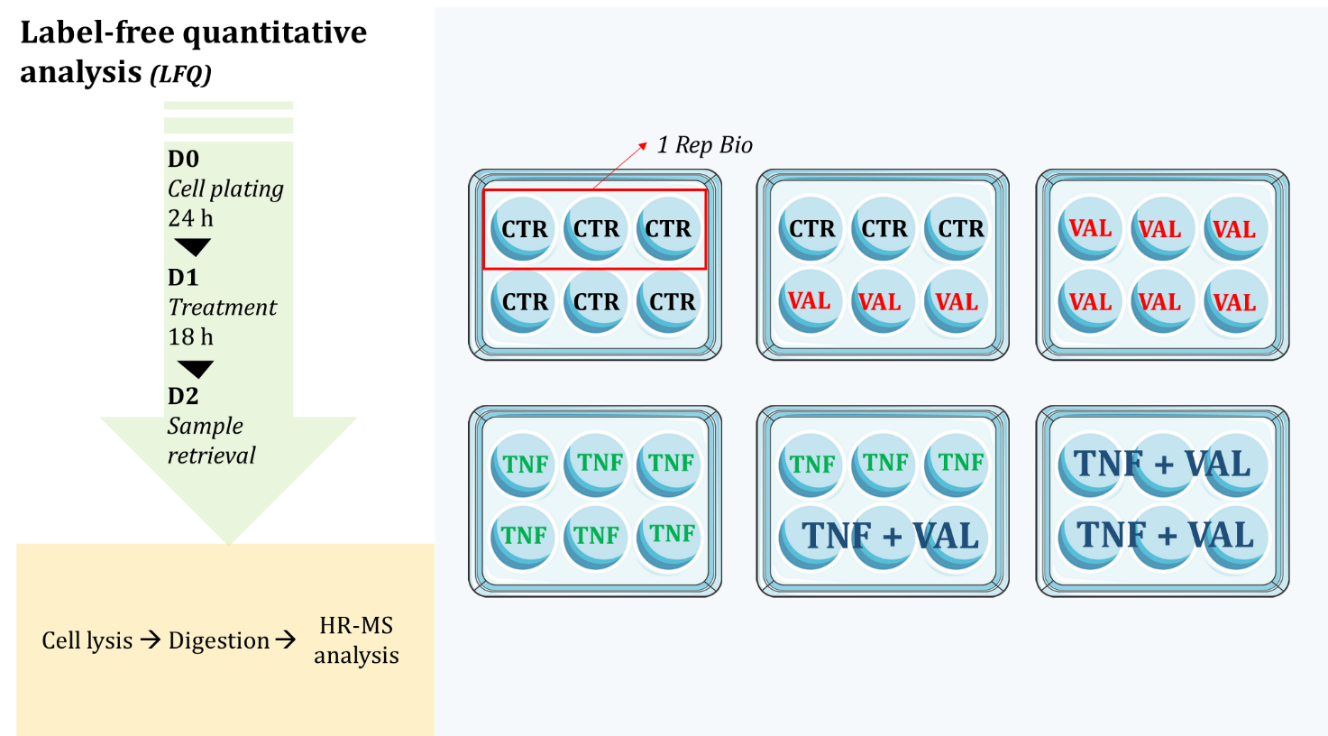
**

**Figure 1 -** Experimental Design of the quantitative proteomic analysis based on LFQ.

**S.6.2 Proteolytic digestion - S-Trap™ Micro Spin Column technology**

Cell lysis was achieved by solubilizing collected pellets in a highly denaturing buffer (5% SDS, 50mM TEAB), supplemented by benzonase (125U/µL) essential for the degradation and elimination of interfering DNA/RNA. Once the lysate was centrifuged and the supernatant pooled, in order to improve proteolytic efficiency, protein mixtures thus extracted were subjected to two additional denaturation steps by acidification and subsequent exposure to a high concentration of methanol.

The reduction of disulfide bridges, alkylation of free thiol residues and sample loading onto the columns were carried out according to the optimized procedure as reported by Ferrario et al. [28]. Upon addition of the protease (1µg sequential grade trypsin, Roche), physical confinement within the pores of the S-trap resin forces the interaction between substrate and protease to yield rapid digestion (1.5 hr, at 47 °C). The peptide mixtures were finally recovered by loading two different elution solutions (hydrophobic conditions) onto spin-columns and dried in the SpeedVac (Martin Christ.) at 37 °C and stored at -80 °C until analysis.

**S.6.3 Data analysis**

*S.6.3.1. STRING analysis*

The STRING software relies on a database of information about known and/or predicted protein-protein interactions. Interactions include direct (physical) and indirect (functional) associations and derived from in silico predictions (e.g., sequence similarities with proteins belonging to different species/organisms). Protein network analysis was performed by setting *Homo sapiens* as the reference database and the confidence cut-off at 0.4 (default); significantly up-regulated proteins having a log2 Fold-Change value > 0.57 and significantly down-regulated proteins with log2 Fold-Change < -0.57 were grouped and examined separately for each experimental condition tested. STRING clusters terms were selected as functional annotations for the enrichment analyses, revealing interesting interactions between equally modulated gene products.

*S.6.3.2. Ingenuity Pathways Analysis (IPA)*

IPA is a bioinformatics software that enables the integration and interpretation of results obtained from proteomics analyses; unlike STRING platform, significantly up- and down-regulated proteins are contextually examined for each experimental condition tested. Using databases and network analysis algorithms, IPA can predict how these interactions may affect cellular processes and metabolic pathways, generating a graphical representation useful for identifying proteins or protein complexes that play an important role in the plausible modulation of a specific pathway. When the causal relationships between the analyzed proteins generate a pattern compatible with that contained within the software, an activation/inactivation hypothesis is generated. The overall activation/inhibition states of canonical pathways are predicted through a z-score algorithm. Activated/inactivated pathways are thus represented and illustrated graphically and may exhibit coloration indicative of their modulation trend: red-orange color predicts activation, while blue predicts inactivation.

**S.6.4. Proteomic studies: results**

**
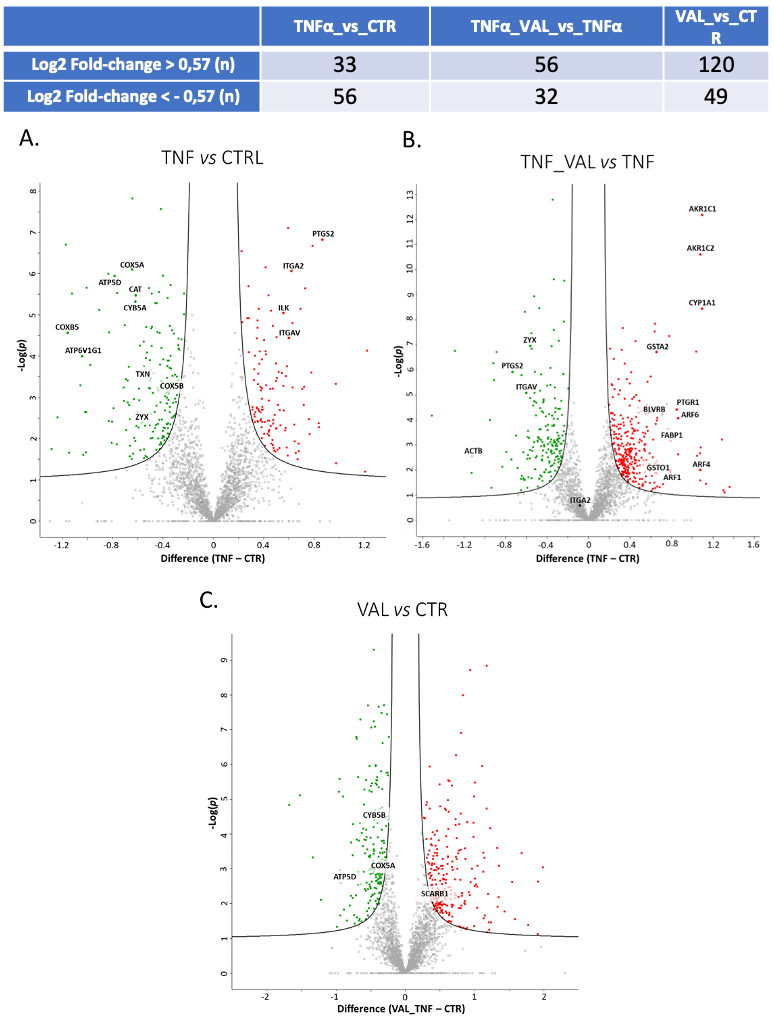
**

**Figure 2** **-** Proteins significantly modulated under the experimental conditions of interest. The upper part of the figure provides a panel of proteins significantly modulated under the experimental conditions of interest (TNFα vs. CTR; TNFα-VAL vs. TNFα; VAL vs. CTR). The lower part of the figure shows the Volcano plots of the LFQ: in the first line, the two Volcano plots (A,B) depict the analyses performed to understand the impact of the TNFα stimulus and the effect of VAL on inflamed cells; in the second line, (C) the Volcano plot shows the effect of VAL on the proteome under physiological conditions. In each volcano plot, the most representative proteins identified in the various analyses are labeled in red if up-regulated (p value < 0.05, log2 Fold-change > 0.57), in green if down-regulated (p value < 0.05, log2 Fold-change < −0.57) and in grey when they do not fall into either of the two aforementioned conditions. For each volcano plot shown, some of the most interesting gene products have been highlighted, the modulation of which will be clarified in the following sections.


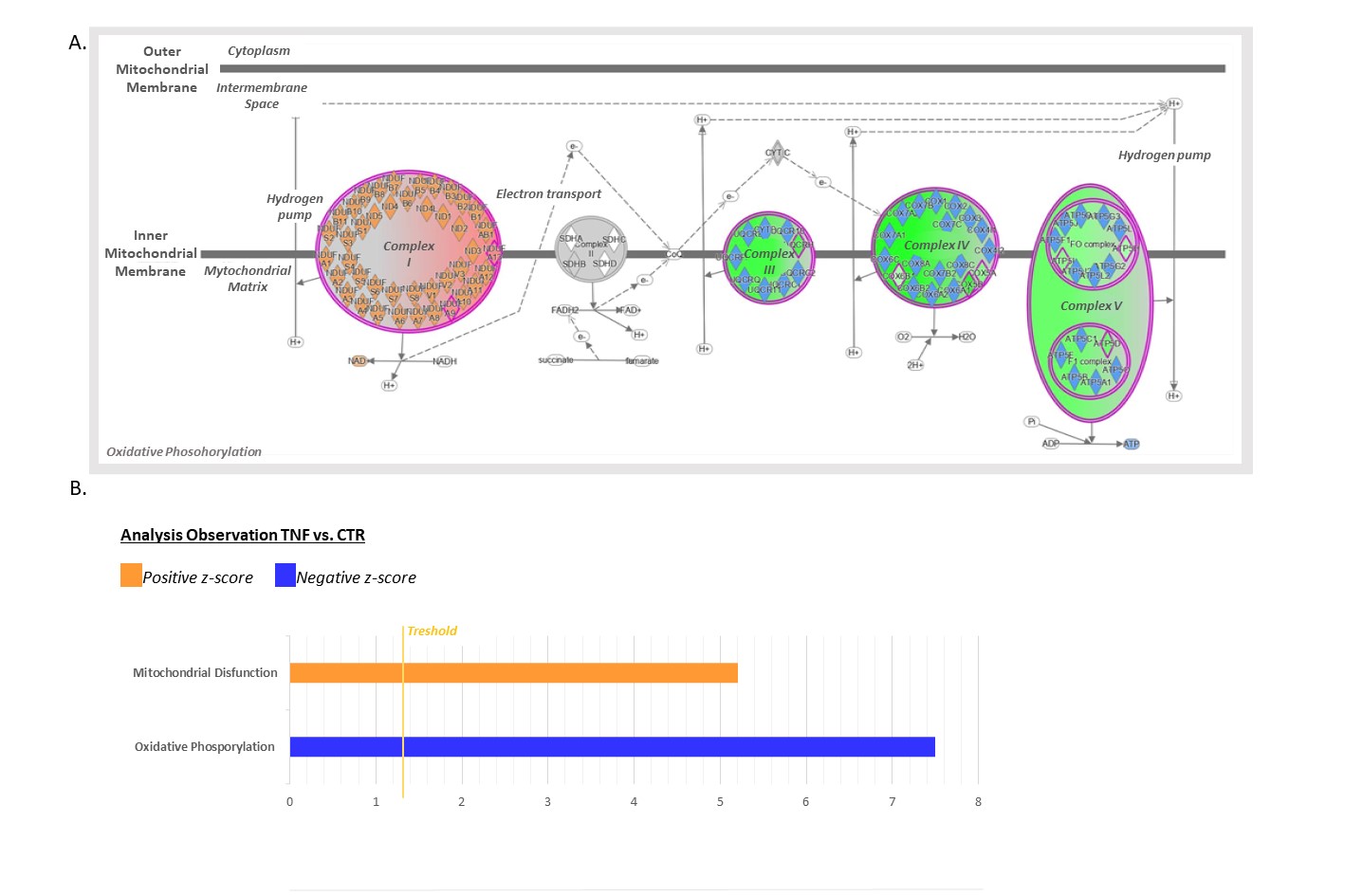


**Figure 3 -** Panel A illustrates the oxidative phosphorylation pathway; in red are shown the proteins that were up-regulated by TNFα and in green those that were down-regulated, overall resulting in activated mitochondrial dysfunction and inhibited oxidative phosphorylation (panel B).

**
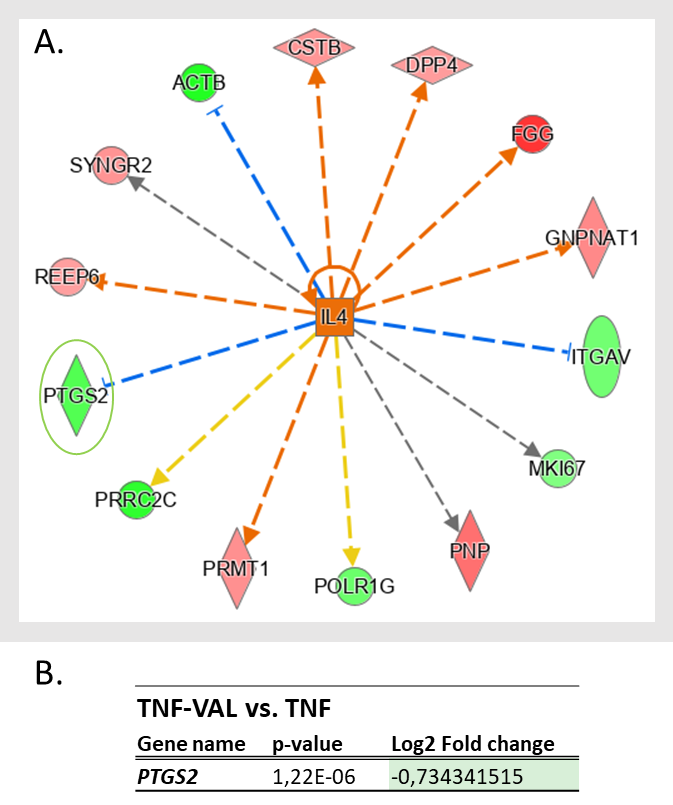
**

**Figure 4 -** Panel A. IL4 was enriched as a potential upstream regulator determined in the TNF-VAL vs. TNF condition by means of IPA analysis (LFQ); the dataset allows a hypothesis of IL4 activation to be generated. PTGS2 is circled in green due to the high interest in the involvement of this protein, whose Log2 fold-change and p-value values are shown in panel B. Color legend: red represents the increased genes, green the decreased (not present in the figure). The intensity of the color is related to the intensity of up- or down-regulation. The orange line leads to activation and a blue line (not present in the figure) leads to inactivation. The yellow line indicates findings that are not consistent with the proteomics results obtained.
